# RNA Metabolism Genes as Prognostic Biomarkers and Therapeutic Targets in Colorectal Cancer Based on the Analysis of Single‐Cell and Bulk‐RNA Sequencing Data

**DOI:** 10.1111/jcmm.71236

**Published:** 2026-06-10

**Authors:** Fandong Kong, Xuewei Zhang, Shuguang Su, Feilong Chen, Qiantao Ye, Ronghua Yang, Hanpeng Du

**Affiliations:** ^1^ Department of Gastrointestinal Surgery Panyu Maternal and Child Care Service Centre of Guangzhou (He Xian Memorial Affiliated Hospital of Southern Medical University) Guangzhou Guangdong China; ^2^ Department of Burn and Plastic Surgery, Guangzhou First People's Hospital Guangzhou Medical University Guangzhou Guangdong China; ^3^ Department of Pathology Panyu Maternal and Child Care Service Centre of Guangzhou (He Xian Memorial Affiliated Hospital of Southern Medical University) Guangzhou Guangdong China

**Keywords:** bulk‐RNA sequencing data, colorectal cancer, prognostic biomarkers, RNA metabolism‐related genes, single‐cell sequencing data

## Abstract

Colorectal cancer (CRC) is one of the most common malignancies worldwide and remains a major cause of cancer‐related mortality. Increasing evidence suggests that aberrant RNA metabolism contributes to tumour initiation, progression and therapeutic resistance. In this study, we integrated single‐cell RNA sequencing and bulk transcriptomic data to identify RNA metabolism‐related genes (RMRGs) associated with CRC progression and evaluate their clinical significance. Through comprehensive bioinformatics analyses, 39 differentially expressed RMRGs were identified, with high RMRG activity predominantly enriched in epithelial cell populations. Cell–cell communication analysis revealed enhanced interactions between epithelial cells and other cell types within the tumour microenvironment. Further integration of single‐cell and bulk RNA‐sequencing datasets identified PCBP3 and NGRN as key prognostic genes. A two‐gene prognostic model based on PCBP3 and NGRN was established and validated in independent cohorts, demonstrating favourable predictive performance. Human Protein Atlas data further confirmed the expression of both proteins in colorectal cancer tissues. Immune infiltration analyses indicated that PCBP3 and NGRN were associated with distinct immune‐cell infiltration patterns and immunotherapy‐related immune status. Functional experiments demonstrated that silencing PCBP3 or NGRN significantly inhibited the proliferation and invasion of HCT116 colorectal cancer cells. Moreover, Western blot analysis suggested that these effects may be mediated, at least in part, through regulation of the PI3K/AKT signalling pathway. Collectively, our findings identify PCBP3 and NGRN as promising prognostic biomarkers and potential therapeutic targets in colorectal cancer and provide new insights into the role of RNA metabolism in colorectal cancer progression and tumour immune regulation.

## 1 Introduction

Colorectal cancer is the third most common cancer worldwide, after lung and breast cancer [1, 2, 3]. Due to the heterogeneity of colorectal cancer, treating colorectal cancer is not a simple medical process [4, 5, 6]. The pathogenesis and evolution of colorectal cancer are not yet fully understood, and research on the treatment of colorectal cancer and the development of related drugs remain challenging. In many areas, the lack of effective screening and predictive biomarkers results in late diagnosis [7, 8]. The early and accurate diagnosis of colorectal cancer is essential for effective treatment and improved patient outcomes [9, 10, 11, 12].

RNA metabolism includes all the processes of synthesis, processing, modification, transport, translation and degradation of RNA molecules [13]. These processes are essential for normal cell function and homeostasis. Dysregulation of RNA metabolism can significantly promote the occurrence and progression of tumours [14, 15]. For example, methyltransferases (such as METTL3) increase m6A modifications [16, 17], which can stabilize carcinogenic mRNA and enhance its translation, promoting tumour growth. Changes in the expression of RNA binding protein (rbp) can stabilize carcinogenic mRNA [18, 19], leading to increased expression of proteins that support tumour growth and survival. Mislocalization of mRNA can lead to inappropriate expression of oncogenes or tumour suppressor genes in normally inactive cell compartmentation, thus promoting tumour development.

Single‐cell sequencing technology can help understand the complexity of tumour heterogeneity and guide the development of more effective combination therapies [20, 21]. Bulk‐RNA helps to profile the tumour microenvironment and identify the different cell types and their interactions that influence tumour growth and metastasis [22, 23]. Single‐cell sequencing (scRNA‐seq) technology allows for the analysis of gene expression at the individual cell level in CRC biology and diagnostic modalities, providing insights that are not possible with bulk sequencing methods.

In this study, the differential expression of RMRGs (DE‐RMRGs) between CRC and normal tissues was measured at single‐cell resolution using bulk‐RNA data, and the expression level of DE‐RMRGs in different cell clusters was investigated, and intercellular communication and locus analysis were performed. By combining large‐volume RNA sequencing data, we constructed a prognostic nomogram using RMRG signatures (PCBP3 and NGRN). We used the GEO dataset to confirm our findings. To investigate the relationship between DE‐RMRGs (PCBP3 and NGRN) and immunoinfiltrating cells in order to understand the potential immunological mechanism of CRC. The correlation between pivot genes and immune checkpoints may provide valuable reference for cancer treatment. In addition, we used NCM‐460 and HCT116 cells to verify gene expression profiles by reverse transcriptional quantitative PCR (RT‐qPCR). This study further explored the effects of PCBP3 and NGRN on the biological behaviour of colorectal cancer cells through in vitro experiments. Our study aims to identify novel biomarkers to improve prediction of colorectal cancer prognosis.

## 2 Materials and Methods

### 2.1 Data Collection and Collation

This study downloaded colorectal cancer datasets from the GEO database [24] (https://www.ncbi.nlm.nih.gov/geo): the microarray sequencing datasets GSE17536 [25] and GSE38832 [26], and the single‐cell sequencing dataset GSE200997. The detailed information of the dataset is presented in Table S1. The sequencing platforms for the GSE17536 and GSE38832 datasets are GPL570, whereas the sequencing platform for the GSE200997 [27] dataset is GPL21697. RNA sequencing data of patients were obtained from TCGA (https://www.cancer.gov/about‐nci/organization/ccg/research/structural‐genomics/tcga). Data sets GSE17536 and GSE38832 were analysed using limma [28] to screen out significant differentially expressed genes (|logFC| > 1 and adjusted *p* < 0.05). GSE200997 single cell sequencing data set, analysis and integration were implemented by R software Seurat (v4.0.6). The normalization and standardization of each sample data were realized by principal component analysis, and the batch difference between samples was realized by Harmony [29] package. The single‐cell data were dimensionalized and visualized by Uniform Manifold Approximation and Projection (UMAP) algorithm. 49,859 single cells were retained and applied for downstream analysis.

### 2.2 Analysis of Tumour Cell Copy Number Variation

Copy number variants (CNVs) are widely used to identify malignant tumour cells in cancer studies [30]. We extracted raw single‐cell gene expression data from Seurat subjects and used publicly available single‐cell data from normal epithelial cells as a control reference [31] (GSE121600). We performed inferred CNV analyses using default parameters. The malignant tumour score was defined as the mean of the square of the final CNVs value on each chromosome, and this score was compared with the standard distribution and checked for bihumped distribution to determine whether it was a malignant tumour or a non‐malignant tumour marker.

### 2.3 Cell Communication Analysis

In order to research the potential between any two different cell types in cell–cell communication, we used R package Cell Chat analyzes ligand receptor (http://www.cellchat.org/index_inner.html) [32]. In the comparison of each pair of cell types, each ligand‐receptor pair will form a null hypothesis distribution based on random substitution. CellChat calculates the *p*‐value of each ligand‐receptor for the likelihood of interaction specific to the cell type by comparing the actual average expression level with this null hypothesis distribution. Finally, the false discovery rate (FDR) method proposed by Yoav Benjamini and Yosef Hochberg was used to adjust the *p*‐value and correct for multiple comparisons of 384 ligand‐receptor pairs and 324 cell pairs.

### 2.4 Gene Enrichment Analysis

We used R‐package clusterProfiler [33] to perform enrichment analysis of GO and KEGG [34] pathways for differentially expressed genes associated with colorectal cancer, in which *p* value < 0.05 and FDR value (*q* value) < 0.25 were used as screening criteria to determine statistical significance.

### 2.5 Construction of Diagnostic Model for Colorectal Cancer

In order to obtain a colorectal cancer (CRC) diagnostic model from a Combined GEO Datasets, we performed Logistic regression analysis of the relevant differentially expressed genes. We further carried out LASSO (Least Absolute Shrinkage and Selection Operator) regression analysis on the differential expression genes. Finally, LASSO RiskScore is calculated based on the risk coefficient of LASSO regression analysis. The formula for calculating risk score is as follows:

$$\text{RiskScore}=\Sigma \;\left(\beta_{i}\times {\text{Gene}}_{i}\right)$$

### 2.6 Validation of Diagnostic Models for Colorectal Cancer

We use R‐package ggDCA to generate a decision curve analysis (DCA) graph for selected Model Genes from a Combined Datasets of GEO [35]. Subsequently, to assess the diagnostic value of the LASSO risk score in predicting the onset of colorectal cancer (CRC), we plotted the ROC curve of the integrated GEO dataset using R package pROC (version 1.17.0.1) and calculated the area under the ROC curve (AUC). Specifically, we obtained patient follow‐up time and gene score expression, performed roc analyses at 365, 1095 and 1825 time points using pROC's ROC function, and evaluated AUC and confidence intervals using pROC's ci function to obtain final AUC results. We further used the R software package survival to integrate survival time, survival status, and gene expression data, and evaluated the prognostic significance of each gene by cox method.

### 2.7 Pan‐Cancer Data Set to Determine the Relationship Between Genes and OS in Different Cancer Species

We downloaded the standardized pan‐cancer dataset from the UCSC (https://xenabrowser.net/) database [36]: TCGA TARGET GTEx (PANCAN, *N* = 19,131, *G* = 60,499). We extracted the expression data of NGRN and PCBP3 in each sample, and screened the sample sources as follows: Primary Blood Derived Cancer—Peripheral Blood (TCGA‐LAML), Primary Tumour, TCGA‐SKCM Metastatic, Primary Blood Derived Cancer—Bone Marrow, Primary Solid Tumour, Recurrent Blood Derived Cancer—Bone Marrow samples. In addition, we also from the TCGA prognosis of previously published in Cell research obtained the prognosis of high‐quality TCGA data set, from the UCSC cancer browser (https://xenabrowser.net/datapages/) to obtain the TARGET follow‐up data. In order to supplement and exclude samples whose follow‐up time was shorter than 30 days, log_2_(*x* + 0.001) transformation was further performed for each expression value. Finally, cancer species with less than 10 samples in a single cancer species were also excluded. We established a Cox proportional hazards regression model using the coxph function of the R package survival (version 3.2‐7) to analyse the relationship between gene expression and prognosis in each tumour, using Logrank test. Further, log_2_(*x* + 0.001) transformation was performed for each expression value, and the gene expression profile of each tumour was extracted separately. The expression profile was mapped to Gene Symbol, and the deconvo_epic method was further used in the R software package IOBR (version 0.99) [37]. The infiltration scores of B cells, CAFs, CD4_Tcells, CD8_Tcells, Endothelial, NK cells and other cells for each patient in each tumour were re‐evaluated according to gene expression. In addition, we also from GDC by MuTect2 [38] (https://portal.gdc.cancer.gov/) to download software all level4 TCGA sample processing Simple Nucleotide for the Variation dataset. We calculated the TMB (Tumour mutation) for each tumour using the TMB function of the R software package maftools (version 2.8.05), we integrated the TMB and gene expression data of the samples, and further carried out log_2_(*x* + 0.001) transformation for each expression value. Finally, we also eliminated the cancer species with < 3 samples in a single cancer species, and finally obtained the expression data of 37 cancer species.

### 2.8 Quasi‐Time Series Analysis

We used the R package monocle3 to perform a single‐cell pseudo‐timing analysis. The dimensionality of the data were reduced by applying the UMAP method, and the result was visualized using the ‘plot_cells’ function. At the same time, the ‘graph_test’ function was used to select differentially expressed genes (DEG). Set the minimum threshold for the Morans index to 0.3 and the maximum threshold for the *q* value (i.e., the corrected *p* value) to 0.001.

### 2.9 Analysis of Transcription Factor Regulation

We use the computational framework PySCENIC to infer the regulatory networks of transcription factors (TFS) from single‐cell RNA sequencing data. Based on the SCENIC (Single‐Cell rEgulatory Network Inference and Clustering) tool, the method is able to reveal how intracellular transcription factors influence cell state and function by controlling the expression of target genes. First, loom files are processed in sparse matrix format, and grnboost2, an algorithm based on Apache Spark, a machine learning framework, is used to identify gene groups with similar expression patterns in the samples and form co‐expression modules for identification. These groups may be regulated by the same transcription factor. Next, DNA‐motif analysis selects potential regulon for TF and uses these co‐expressed gene groups to deduce possible transcription factors and their target genes through dynamic analysis of binding sites rich in known transcription factors in gene promoter regions. Finally, using AUCell will assess the regulatory intensity of individual transcription factors corresponding to target genes and build regulatory networks for each cell type or state based on this information.

### 2.10 Immunoinfiltration Analysis

We combined the data uploaded to focus on the gene expression of matrix CIBERSORTx online platform (https://cibersortx.stanford.edu/), and use LM22 feature gene matrix is analysed. By screening samples with *p* values < 0.05, we obtained a preliminary immune cell infiltration matrix. Further, we only retained data with an immune cell enrichment fraction greater than zero, resulting in a detailed matrix of immune cell infiltration. Finally, the infiltration levels of these 22 immune cells were visualized to show the distribution of each cell type.

### 2.11 Cell Culture and Transfection

NCM‐460 and HCT116 cells were cultured in Dulbecco's Modified Eagle Medium (DMEM) (Xiamen Immocell Biotechnology Co. Ltd., China), supplemented with 10% fetal bovine serum (FBS) and 1% penicillin–streptomycin (PS) (Xiamen Immocell Biotechnology Co. Ltd., China). The culture conditions were maintained at 37°C under 5% CO_2_. Subsequently, HCT116 cells were transfected with small interfering RNA (siRNA) targeting PCBP3 and NGRN (Gemma Gene, Suzhou, China). The specific sequences of the siRNA used in this study are detailed in Table S1.

### 2.12 Real‐Time PCR

Extract total RNA from samples using TRIzol reagent (Invitrogen, Thermo Fisher Scientific Inc., Waltham, USA). Convert RNA to cDNA using reverse transcription with specific primers and reverse transcriptase (Vazyme Biotech Co., Nanjing, China). Prepare a master mix containing SYBR Green (Vazyme Biotech Co., Nanjing, China), primers and buffer. Add cDNA samples. Run the PCR machine with cycles of denaturation, annealing and extension. The relative messenger RNA (mRNA) expression levels were eventually enumerated using the $2^{-∆∆C_{t}}$ method against GAPDH as the internal standard. The primers were provided by Sangon Biotech Inc. Ltd. (Shanghai, China). The detailed steps and procedures for reverse transcription and qRT‐PCR analysis are displayed in Tables S3 and S5.

### 2.13 CCK‐8 Assay

Seed cells in 96‐well plates at desired densities. Incubate plates under standard conditions until cells reach the desired growth phase. Aspirate media and add CCK‐8 solution (Beyotime Biotechnology Inc. Ltd., Shanghai, China) to each well. Incubate plates for a specified duration to allow formazan formation. Read absorbance at 450 nm using a microplate reader. Calculate cell viability based on absorbance values relative to controls.

### 2.14 Clone Forming Assay

Seed HCT116 cells at a density of 800 cells per well into a 12‐well plate, and culture them for the designated time period with regular medium changes. After 2 weeks of incubation, carefully wash the cells with PBS, then fix them with 10% formaldehyde at room temperature for 15 min. Subsequently, stain the cells with crystal violet (Beyotime Biotechnology Inc. Ltd., Shanghai, China) for 15 min. Finally, count the formed cell colonies using an optical microscope (Olympus, Tokyo, Japan).

### 2.15 Transwell Assay

Prior to conducting the Transwell assay, adjust the cell density to 5 × 10^5^ cells/mL. Introduce 200 μL of the pre‐adjusted cell suspension in serum‐free medium into the upper chamber of the Transwell to assess migration capability. After incubating for 12–48 h under standard conditions in the incubator, fix the wells with methanol for 30 min. Subsequently, stain the cells with 0.1% crystal violet (Beyotime Biotechnology Inc. Ltd., Shanghai, China) for 20 min. Observe the cells under the microscope. Finally, count the positively stained cells under the microscope.

### 2.16 Statistical Analysis

Using R software (v4.3.1) for statistical analysis, we performed a *T*‐test for comparison between groups to analyse differences in normally distributed variables. For variables that are not normally distributed, we use the Mann–Whitney *U* test, also known as the Wilcoxon Rank Sum Test, to assess the difference between them. When three or more groups are involved, the Kruskal‐Wallis test is used for analysis. All of these results were correlated using Spearman correlation analysis to calculate the correlation coefficients between different molecules.

## 3 Results

### 3.1 Identification of Tumour Cells and Acquisition of Differential Genes

**FIGURE 1:**
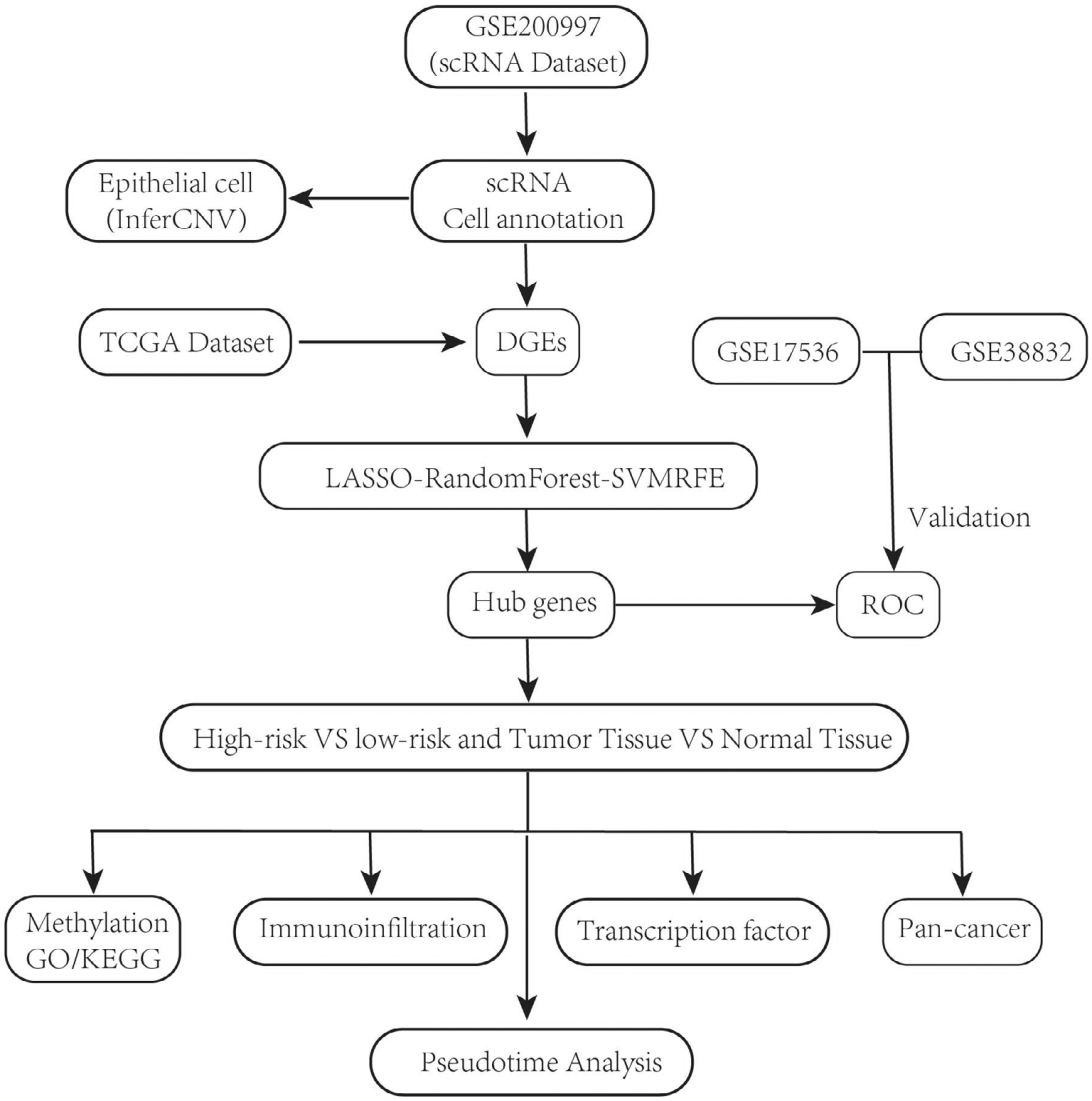
The workflow of the study.

**FIGURE 2:**
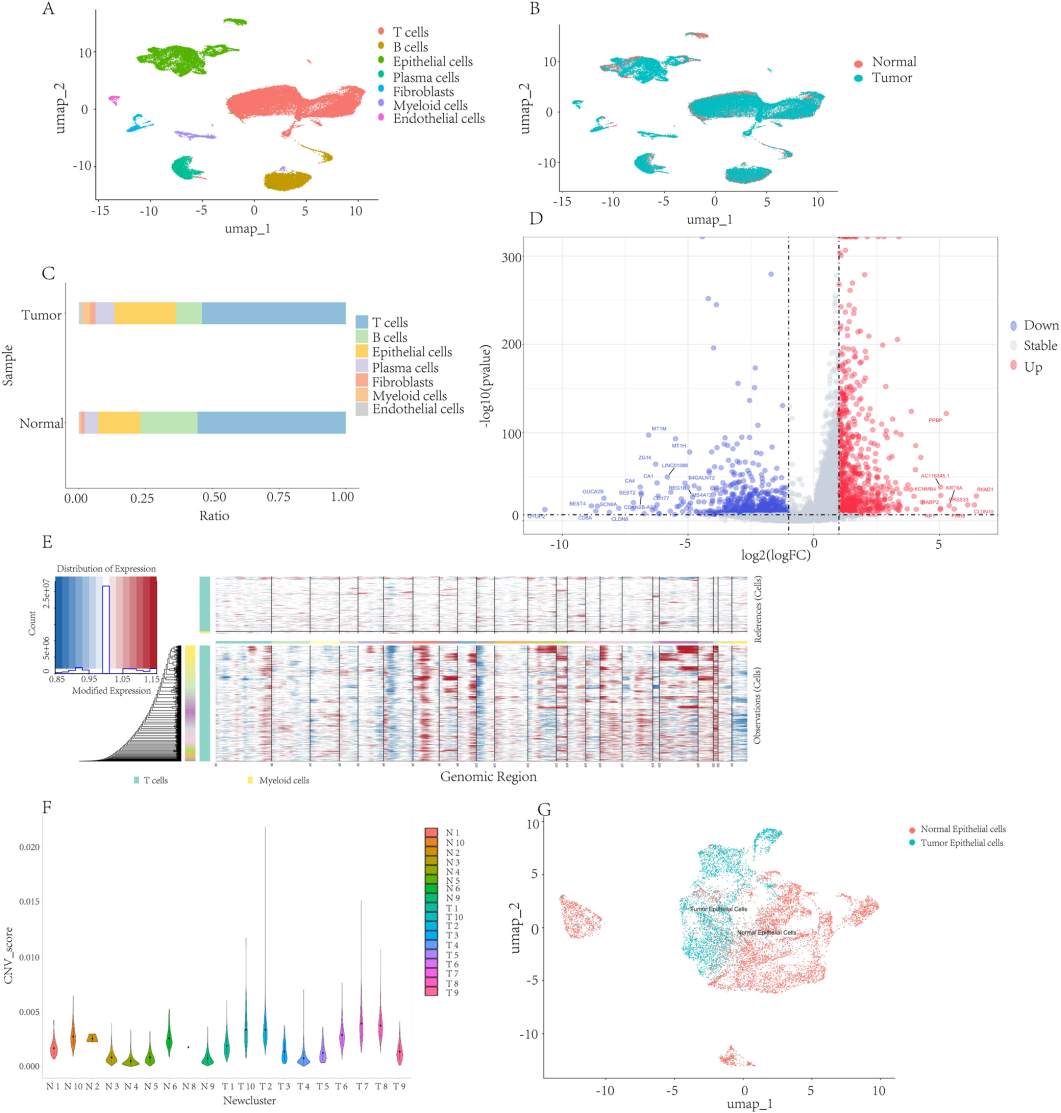
Cell type identification by scRNA‐seq analysis of colorectal cancer. (A, B) Uniform manifold approximation and projection (UMAP) plot showing the seven cell types of normal tissue and tumour tissue of colorectal cancer patients. (C) Bar plot showing the proportions of different cells in normal and tumour tissues. (D) Volcano plot showing upregulated and downregulated genes in tumour tissue, with |logFC| > 1 and *p* > 0.05. (E) CNV heatmaps showing expression data generated by infercnv analysis. (F) Violin plot showing the CNV scores in different clusters. (G) UMAP plot showing the distribution of tumour epithelial cells obtained after inferCNV analysis.

Figure 1 is a description of the technology roadmap. The data sets were analysed using standard single‐cell RNA sequencing data processing. First, the UMAP technology was used to reduce the dimensionality of the data, and the UMAP map (Figure 2A) was generated, which visually demonstrated the cell clustering in the data. Through in‐depth annotation of the cells in the UMAP, we identified the following cell types: T cells, B cells, epithelial cells, plasma cells, fibroblasts, myeloid cells and endothelial cells. Comparing cells from different tissue sources, we found that the proportion of epithelial cells in tumour tissues was significantly higher than that in normal tissues (Figure 2B). In addition, by visualizing tissue source information with UMAP again (Figure 2C), it was confirmed that cell annotation is based on cell clustering rather than tissue source. Based on differential gene expression, a volcano map was generated (Figure 2D), which clearly showed genes that were expressed differently in epithelial cells. The copy number variation (CNV) of all epithelial cells was then analysed using inferCNV, with T cells and myeloid cells serving as reference populations. The CNV heatmap revealed that specific epithelial‐cell clusters (Clusters 2, 7, 8 and 10) in tumour tissues exhibited the highest CNV levels (Figure 2E). Quantitative comparison of CNV scores across different clusters further confirmed these findings (Figure 2F). Based on the inferCNV results, Clusters 2, 7, 8 and 10 were defined as tumour epithelial cells. The distribution of tumour epithelial cells identified by inferCNV analysis was subsequently visualized using UMAP (Figure 2G), further supporting their malignant characteristics and distinguishing them from normal epithelial cells.

### 3.2 Screening of the Most Appropriate Differential Genes Affecting Prognosis

**FIGURE 3:**
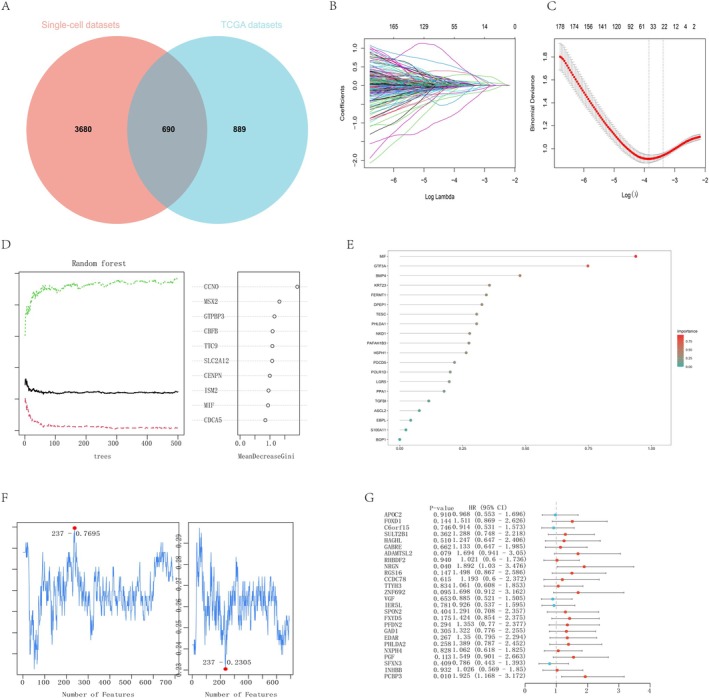
Screening of disease intersection genes and machine learning algorithm to identify the optimal feature genes. (A) Upregulated genes in single‐cell dataset and Limma analysis of differential genes in TCGA CRC data were used to do a Venn diagram screening of intersecting genes. (B, C) LASSO coefficient profiles of the candidate optimal feature genes, and the optimal lambda was determined when the partial likelihood deviance reached the minimum value. Each coefficient curve in the left picture represents a single gene. The solid vertical lines in the right picture represent the partial likelihood deviance and the number of genes (*n* = 35). (D) Relative importance of overlapping candidate genes calculated in random forest (Top 10 genes' importance > 2). Importance indexes on the *x*‐axis and genetic variables are plotted on the *y*‐axis. (E) Relative importance of overlapping candidate genes calculated in random forest. Importance indexes on the *x*‐axis and genetic variables are plotted on the *y*‐axis. (F) The SVM‐RFE algorithm was used to further identify candidate optimal feature genes, with the highest accuracy and lowest error obtained in the curves. The *x*‐axis shows the number of feature selections, and the *y*‐axis shows the prediction accuracy. Sixteen gene features were identified through SVM‐RFE analysis with an accuracy of 0.7695 and an error of 0.2305. (G) Forest plot showing multivariate Cox proportional hazards regression analysis in TCGA CRC dataset with filtered genes.

First, we analysed the 4370 up‐regulated genes of tumour epithelial cells in single‐cell sequencing and the Venn map of 1579 up‐regulated genes in the tumour group in the TCGA database through limma package, and finally obtained 690 intersection genes (Figure 3A). This result points to a significant intersection between up‐regulated genes observed in different datasets that may play a key role in tumour development and progression. Following Lasso regression analysis of 690 intersection genes, 39 genes were found to be significantly associated with patient outcomes (Figure 3B,C). This analysis helped us narrow down the genes to focus on, setting the stage for further validation and functional studies. The importance of these genes to prognosis was subsequently assessed by random forest analysis, which further validated the results of the Lasso regression analysis and revealed which genes were likely to be of higher biological importance in the prognosis assessment (Figure 3D,E). At the same time, using the support vector machine‐recursive feature elimination (SVM‐RFE) method, we selected 238 optimal feature genes with the lowest error and the most significant feature. The identification accuracy of these genes was 0.7695, and the error rate was 0.2305, indicating that this method has high efficiency and accuracy in screening genes closely related to prognosis (Figure 3F). Finally, we evaluated all genes by single‐gene and multigene cox regression analyses. The display and analysis of forest maps identified PCBP3 and NGRN as the genes with the greatest prognostic impact, a finding that provides potential targets for future targeted therapies against these genes (Figure 3G).

### 3.3 The Gene Pair Prognosis Model Composed of PCBP3 and NGRN Can Be Used as Potential Biomarkers for Cancer Prognosis

By analysing the polygenic Cox regression model constructed from the TCGA dataset using the machine‐learning strategies described above, we selected PCBP3 and NGRN as a gene pair for prognostic prediction, with the aim of identifying potential biomarkers for clinical application. The expression profiles of PCBP3 and NGRN were incorporated into a multigene Cox regression model, which was subsequently validated using both TCGA and GEO datasets.

**FIGURE 4:**
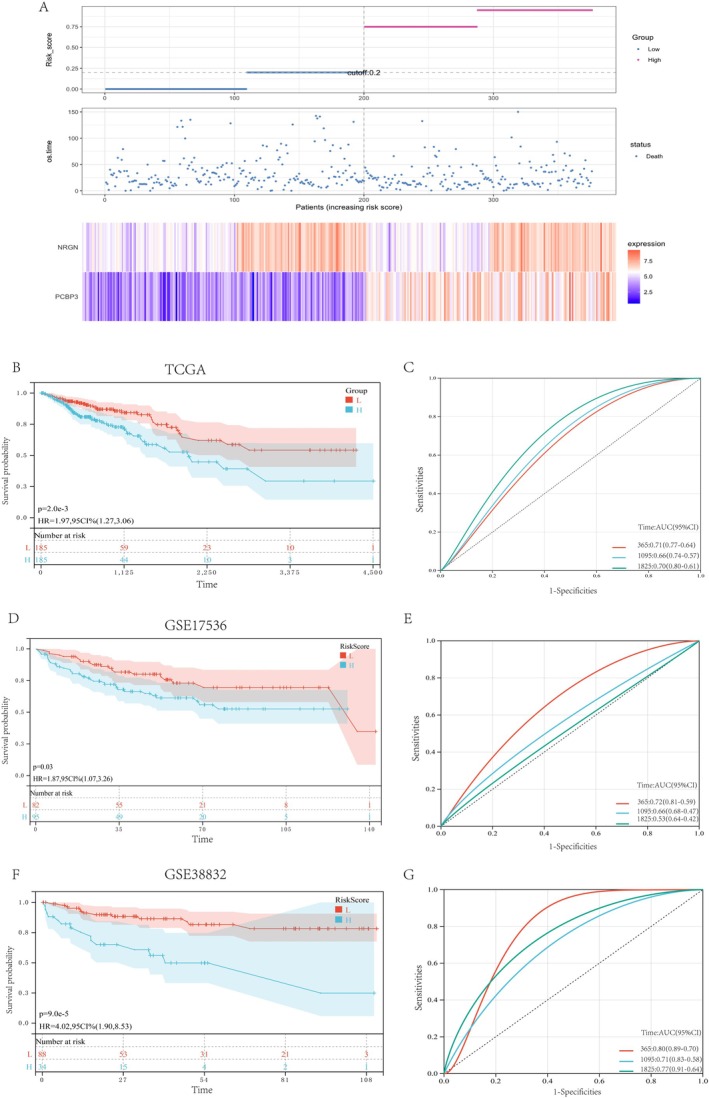
Construction of a risk score model using PCBP3 and NGRN. (A) The image showing the analysis of factors associated with immune scoring and survival analysis results. (B) Survival curves of risk scores in the TCGA database. (C) ROC curves of the ability of risk score to predict 1‐, 2‐ and 3‐year overall survival in TCGA dataset. (D) Survival curves of risk scores in the GSE17536 database. (E) ROC curves of the ability of risk score to predict 1‐, 2‐ and 3‐year overall survival in GSE17536 database. (F) Survival curves of risk scores in the GSE38832 database. (G) ROC curves of the ability of risk score to predict 1‐, 2‐ and 3‐year overall survival in GSE38832 dataset.

First, the optimal cutoff value of the Cox regression model was calculated, and 0.2 was identified as the most appropriate threshold. The relationship between risk score and overall survival time was then visualized, allowing direct observation of the association between increasing risk score and poorer patient outcome. Heatmap analysis further showed that the expression levels of PCBP3 and NGRN increased with elevated risk scores (Figure 4A). After model construction, the TCGA, GSE17536, and GSE38832 datasets were used for validation. In the TCGA cohort, the survival difference between the two risk groups was statistically significant (*p* = 2.0 × 10^−3^), and the first‐year AUC reached 0.71, indicating good predictive performance (Figure 4B,C). In the GSE17536 dataset, the AUC values at 1, 3 and 5 years were 0.72, 0.66 and 0.53, respectively, suggesting relatively strong early predictive accuracy that gradually declined over time (Figure 4D,E). In the GSE38832 dataset, the AUC values at 1, 3 and 5 years were 0.80, 0.71 and 0.77, respectively, and the Kaplan–Meier analysis also showed strong statistical significance (*p* = 9.0 × 10^−5^), further supporting the reliability of the model (Figure 4F,G). Collectively, these findings indicate that the PCBP3/NGRN gene pair has robust prognostic value in colorectal cancer across multiple transcriptomic cohorts.

To further strengthen model validation at the protein level, representative immunohistochemical staining images of PCBP3 and NGRN in colorectal cancer tissues were obtained from the Human Protein Atlas (HPA) database. As shown in Figure S1, both proteins were detectable in colorectal cancer tissues, with staining intensities ranging from not detected to high. This orthogonal validation extends the evidence beyond transcriptomic datasets and provides additional support for the biological relevance of PCBP3 and NGRN as candidate prognostic biomarkers in colorectal cancer.

### 3.4 Comparative Analysis of Methylation Differences and Influencing Gene Differences in High and Low Risk Groups

**FIGURE 5:**
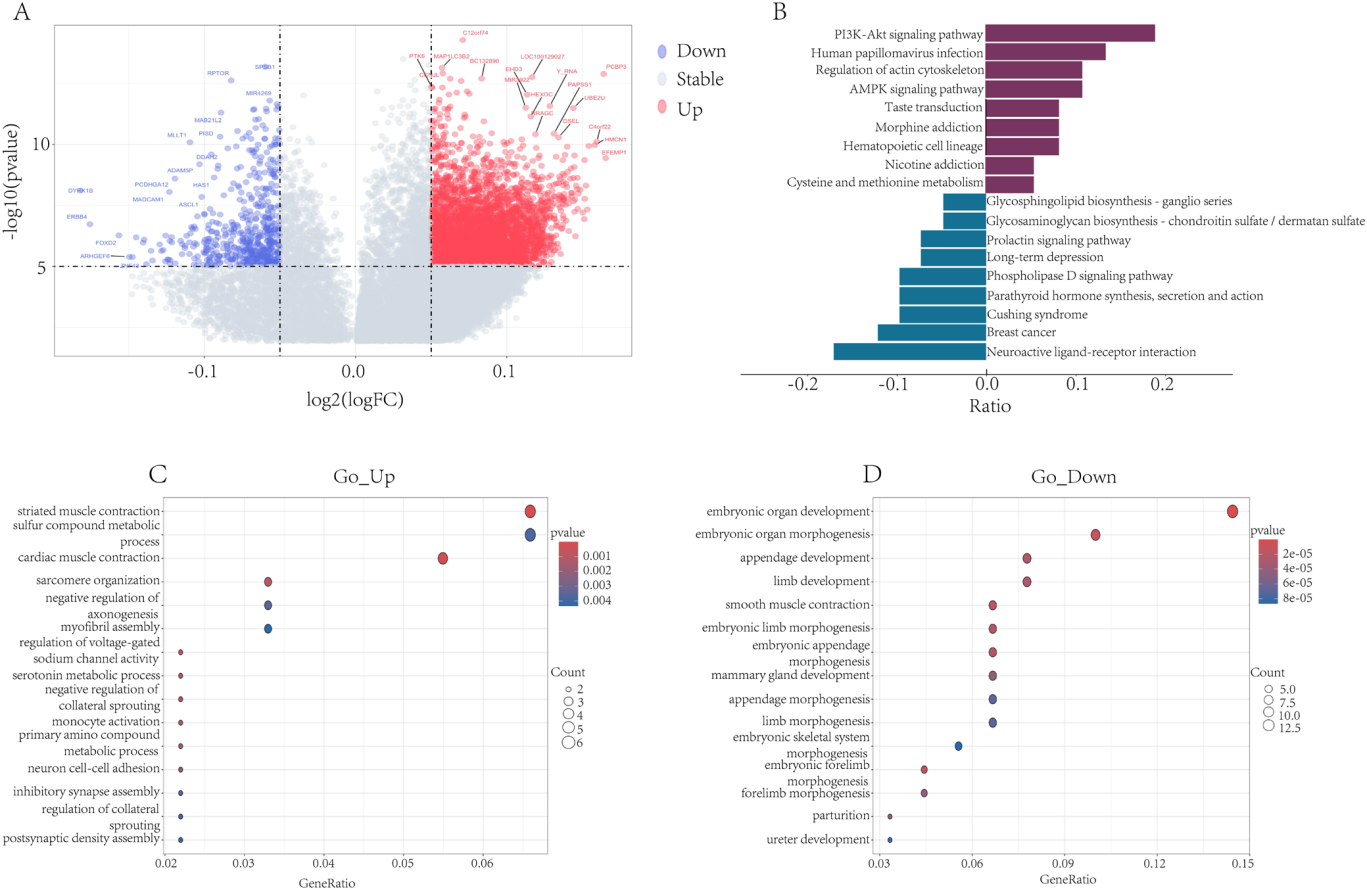
Different patterns of genomic and methylation changes in high and low risk groups. (A) Volcano plot showing upregulated and downregulated genes associated with methylation sites, with |logFC| > 1 and *p* < 0.05. (B) Horizontal bar plot showing the KEGG functional enrichment analysis of differential genes. (C, D) Bubble plot showing the GO functional enrichment analysis of differential genes.

We focused on the models of genes PCBP3 and NGRN, and used the TCGA database methylation data analysis to explore the effects of methylation on gene function and pathways between high and low risk groups. First, we showed the differential expression of the extracted genes in the high‐risk and low‐risk groups, and converted the methylation sites into corresponding genes according to the mapping data set provided by GPL16304. The volcano map distinguished the two groups by red and blue dots, red representing genes in the high‐risk group and blue representing genes in the low‐risk group (Figure 5A). The up‐regulated genes are mainly concentrated in the ‘PI3K‐Akt signaling pathway’ (PI3K‐Akt signalling pathway) and the ‘AMPK signaling pathway’ (AMPK signalling pathway), which play a key role in cell survival and metabolic regulation. Down‐regulated genes are enriched in the ‘Neuroactive ligand‐receptor interaction’ and ‘Breast Cancer’ pathways, suggesting that alterations in these pathways may be involved in the development of colon cancer (Figure 5B). The chart helps to quickly identify genes whose expression differs significantly between the two groups. Subsequently, GO (Gene Ontology) analysis of differential genes showed functional enrichment. The gene functions affected by methylation were mainly concentrated in ‘striated muscle contraction’ (striated muscle contraction), ‘sulur compound metabolic process’ (sulfide metabolic process) and ‘cardiac muscle contraction’ (contraction of the heart muscle) and other biological processes. This suggests that these processes may have a more important role in the high‐risk group (Figure 5C). And methylation affects the functional enrichment of down‐regulated genes, Emphasis is placed on ‘embryonic organ development’ (embryonic organ development), ‘embryonic organ morphogenesis’ (embryonic organ morphogenesis), and ‘appendage development’. Downregulation of these functions may have implications for cancer progression (Figure 5D). Finally, KEGG (Kyoto Encyclopedia of Genes and Genomes) analysis revealed the distribution of up‐regulated and down‐regulated genes in different biological pathways.

### 3.5 Comparative Analysis of Immune Infiltration Differences Between High and Low‐Risk Groups

**FIGURE 6:**
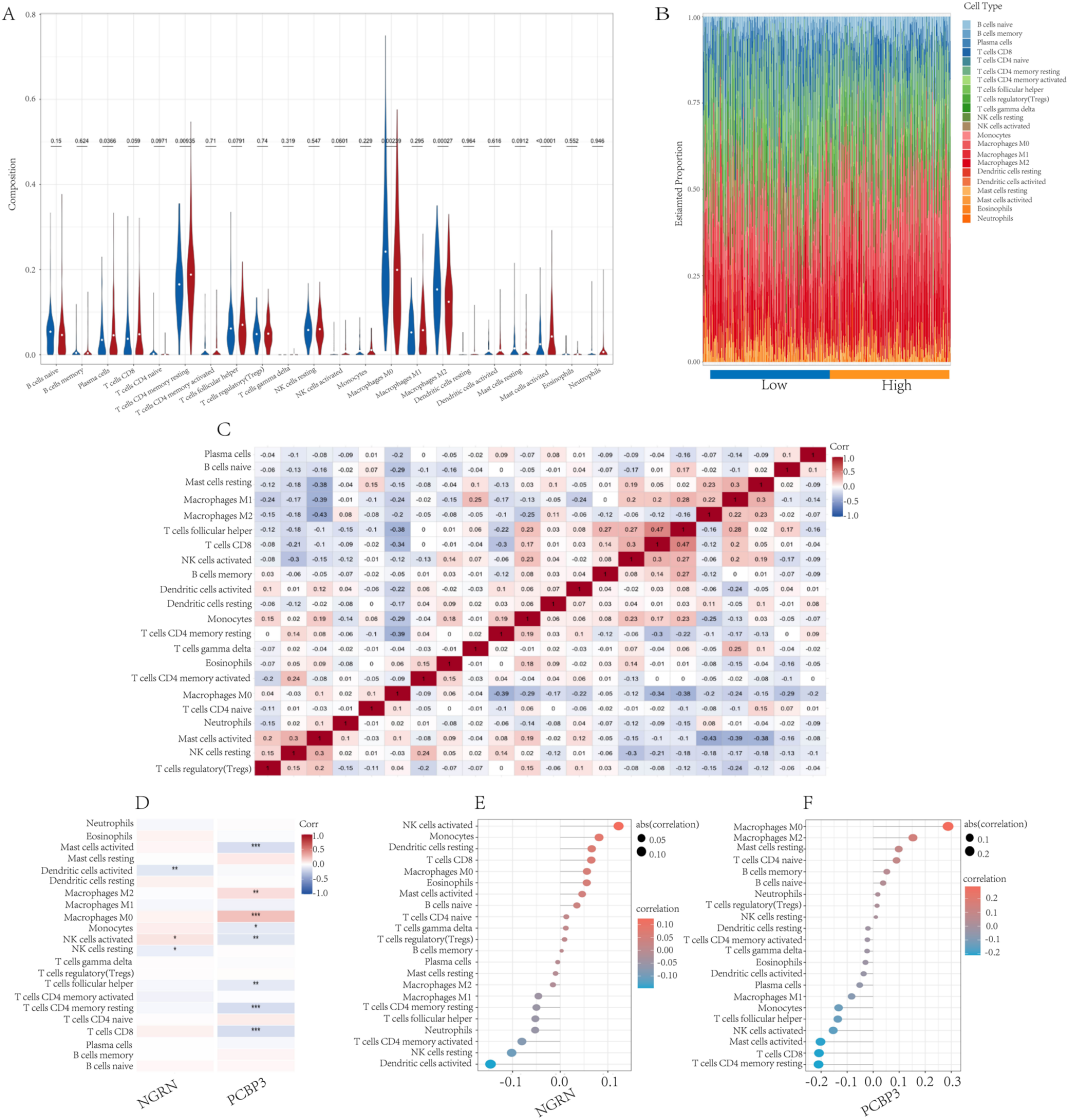
Differences in immune cell infiltration between high and low risk groups in immune infiltration analysis. (A) Violin plot showing the differences in types of immune cells between high and low risk groups. (B) Heatmap showing the differences in immune cell proportions between high and low risk groups. (C) The relative proportions of 22 immune cell types between high and low risk groups in TCGA CRC dataset. (D–F) Correlation analysis of 2 related disease genes and associated immune cells. *p < 0.05, **p < 0.01, ***p < 0.001.

We investigated the prognostic relevance of the PCBP3/NGRN‐related risk model in colorectal cancer by focusing on its association with immune‐cell infiltration in the TCGA cohort using the CIBERSORT algorithm. Initially, the violin plot showed the distribution of 22 immune‐cell types in the two risk groups and revealed significant differences in Macrophages M0 and Macrophages M2 (*p* = 0.00239 and *p* = 0.00027, respectively), suggesting their potential involvement in immune escape and tumour‐microenvironment remodelling in colorectal cancer (Figure 6A). Furthermore, heatmap analysis illustrated the differences in immune‐cell proportions between the high‐ and low‐risk groups (Figure 6B), while the correlation heatmap demonstrated the interaction patterns among different immune‐cell populations, indicating potential shared functions or regulatory relationships (Figure 6C). In terms of the association with the prognostic gene pair, NGRN was primarily correlated with NK cells and dendritic cells, whereas PCBP3 was mainly associated with Macrophages M0/M2 and mast cells, suggesting that these genes may influence colorectal cancer prognosis through distinct immune‐related pathways (Figure 6D). We further visualized the strength of these associations using lollipop plots (Figure 6E,F). Collectively, these findings indicate that the PCBP3/NGRN‐related signature is closely linked to differences in immune infiltration patterns in colorectal cancer.

To further evaluate whether these immune‐cell variations were associated with immunotherapy‐relevant immune status, we additionally performed an IPS‐based analysis in the same cohort. As shown in Figure S3, the IPS_High group exhibited significantly lower PCBP3 expression (*p* = 0.0026) and lower NGRN expression (*p* = 0.011) than the IPS_Low group. In parallel, the IPS_High group showed significantly lower infiltration of Macrophages M0 (*p* = 1 × 10^−5^) and Macrophages M2 (*p* = 0.0067), together with significantly higher levels of activated NK cells (*p* = 0.0032). These results suggest that lower PCBP3/NGRN expression is associated not only with altered immune infiltration, but also with a more immunologically active tumour microenvironment and a potentially more favourable immunotherapy‐related immune status in colorectal cancer.

### 3.6 Analysis of NGRN and PCBP3 in Different Cancer Species

**FIGURE 7:**
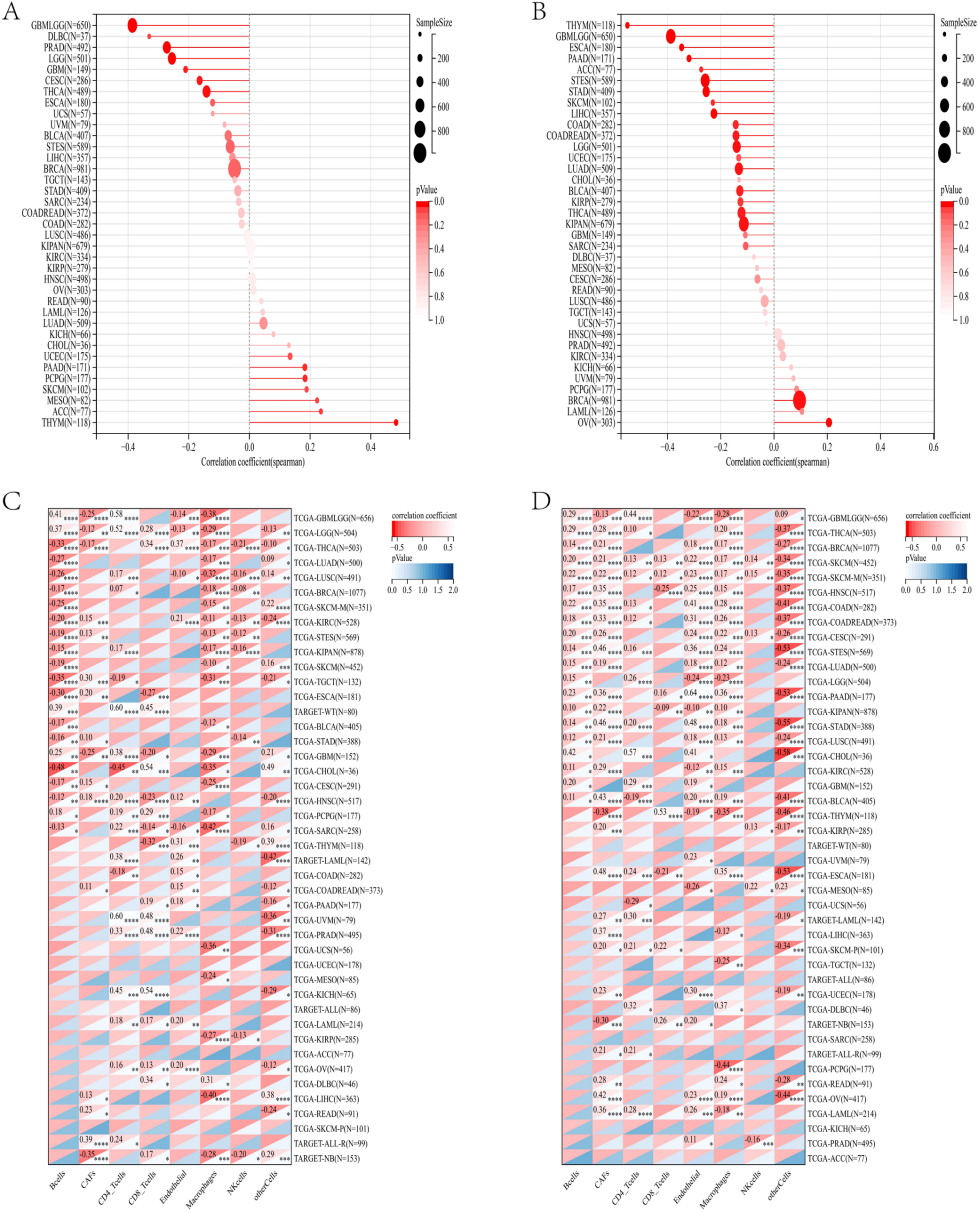
Immune cell analysis and genomic heterogeneity analysis of pan‐oncogenes. (A) Heterogeneity and expression analysis of NGRN. (B) Heterogeneity and expression analysis of PCBP3. (C) Immunocell analysis of NGRN. (D) Immunocell analysis of PCBP3. *p < 0.05,**p < 0.01,***p < 0.001.

**FIGURE 8:**
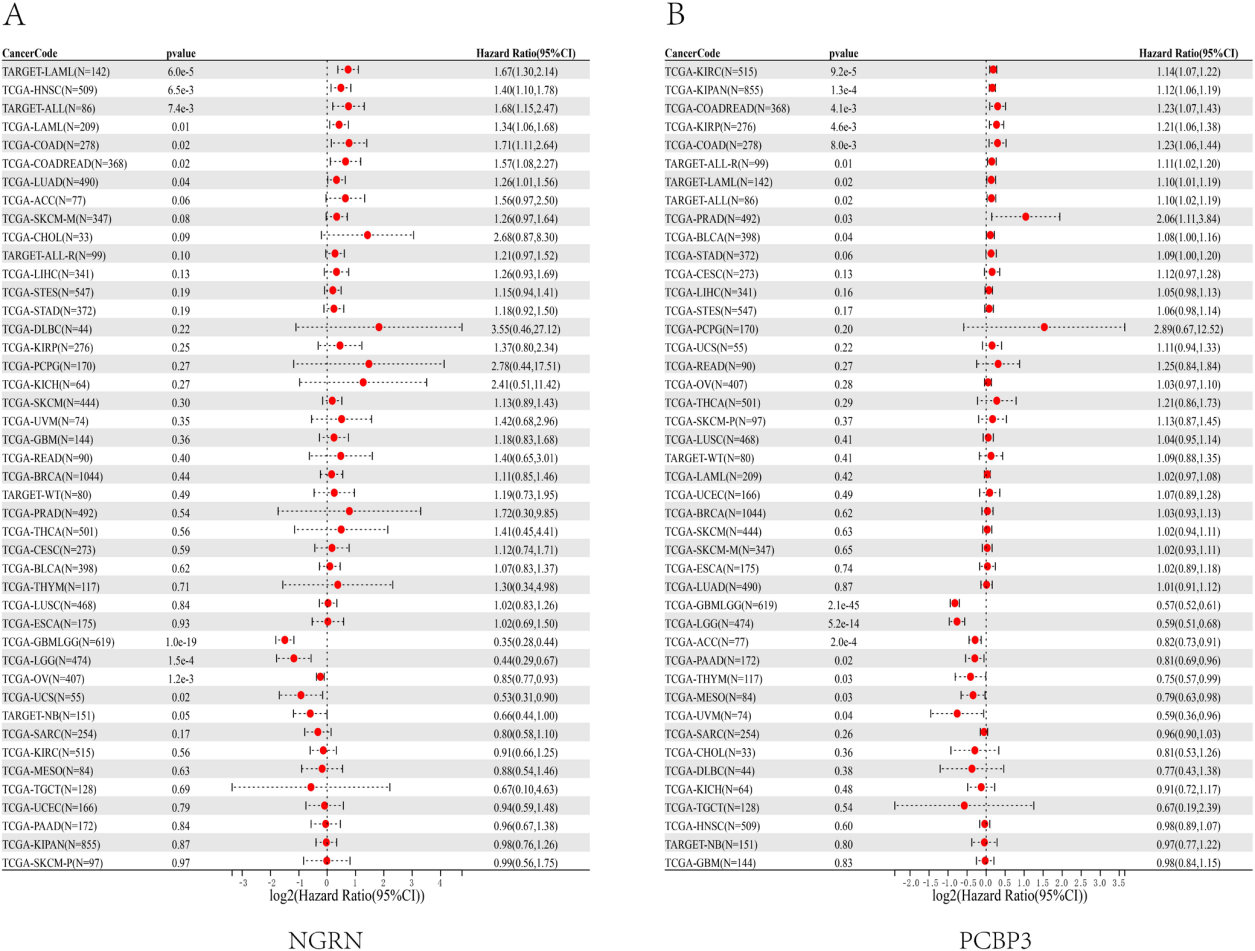
Prognosis analysis of pan‐oncogene expression. (A) Prognostic analysis of NGRN and pancarcinoma. (B) Prognostic analysis of PCBP3 and pancarcinoma.

We further obtained 8 categories of immunocell infiltration scores for 10,180 tumour samples from 44 tumour types. Pearson's correlation between gene and immunocell infiltration scores in each tumour was calculated using the corr.test function of the R package psych (version 2.1.6) coefficient, to determine the immunoinfiltration score of significant correlation. Their spearman correlation was calculated in each tumour, and for NGRN we observed a significant association in 12 tumours, with a significant positive association in 5 tumours and a significant negative association in 7 tumours including CRC (Figure 7A). In the PCBP3 analysis, we observed a significant association in 19 tumours, with a significant positive association in 2 tumours and a significant negative association in 17 tumours, including CRC (Figure 7B). NGRN analysis showed that the expression of this gene was significantly correlated with immunoinfiltration in 40 cancer species (Figure 7C), and in 39 cancer species in PCBP3 (Figure 7D). Then, through the extensive cancer data set of NGRN OS analysis shows, eventually won the 44 carcinoma of expression data and corresponding sample overall survival data, finally observed in seven tumour types (LAML LUAD, COAD, COADREAD, HNSC, LAML, high expression of ALL had poor prognosis, and low expression of ALL) had poor prognosis in 5 tumour types (GBM, LGG, NB, OV, UCS), as shown in Figure 8A. OS analysis of PCBP3 showed that 10 tumour types (LAML, KIRP, KIPAN, COAD, COADREAD, PRAD, KIRC, etc.) were finally observed. High expression in BLCA, ALL, ALL‐R had poor prognosis, whereas low expression in 7 tumour types (GBMLGG, LGG, THYM, MESO, UVM, PAAD, ACC) had poor prognosis (Figure 8A,B).

### 3.7 Analysis of Cell Interactions and Transcription Factor Regulatory Networks

**FIGURE 9:**
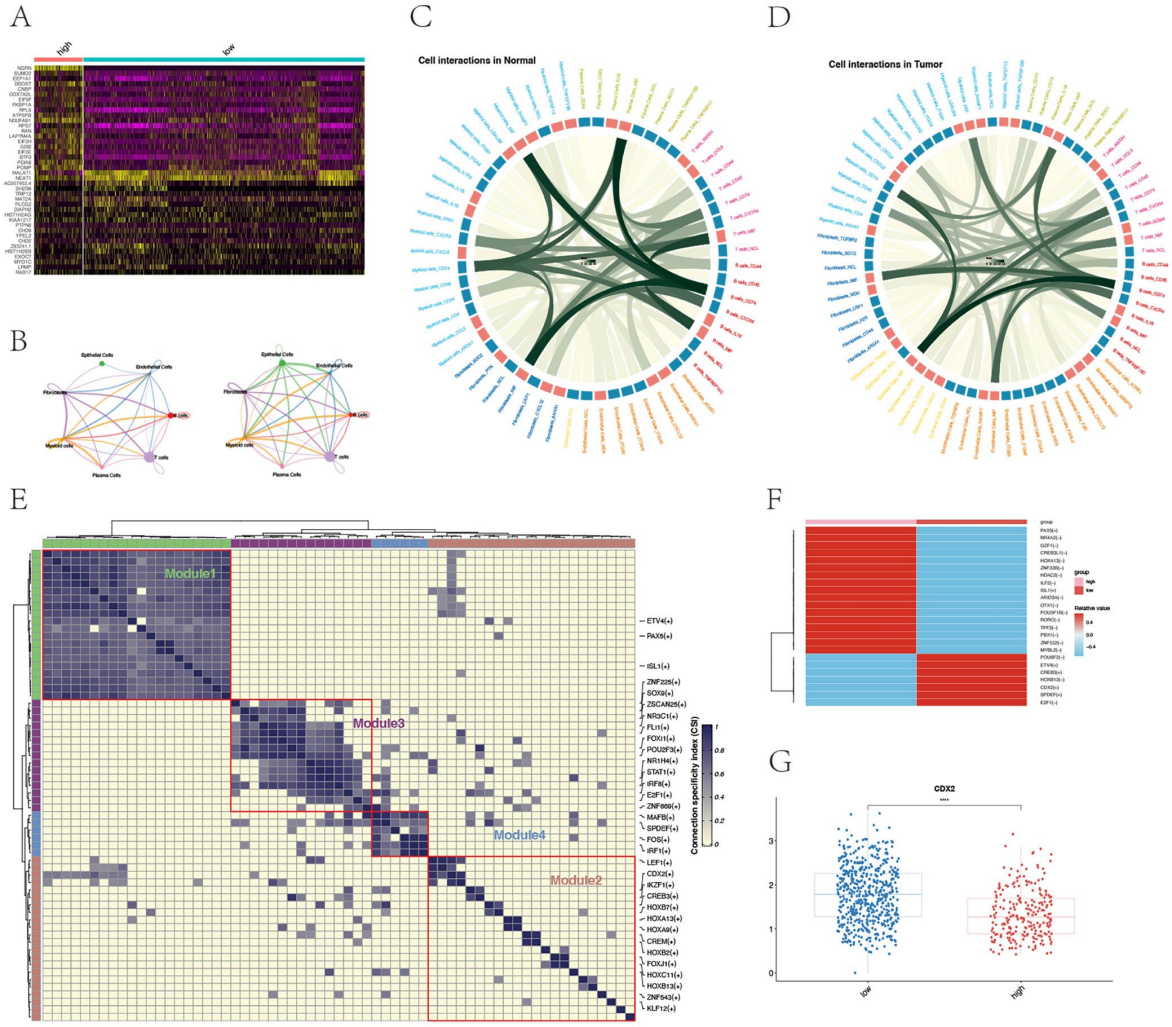
Differences in cell interactions between high and low risk groups. (A) Heatmap showing the differential genes between high and low risk groups. (B–D) Chord diagram showing the differences in cell interactions between high and low risk groups. (E) Heatmap showing the status of the transcription factor module in the overall tissue. (F) Heatmap showing the differences of the transcription factor module between high and low risk groups. (G) Box plot showing the expression differences of the CDK2 gene between the two groups. *p < 0.05, **p < 0.01, ***p < 0.001.

The risk grouping of single‐cell data were evaluated according to the risk factor model obtained from the analysis of transcriptomic data, and the array above the median risk factor was defined as ‘high’ and the array below the median risk factor was defined as ‘low’ to explore the differences in cell interactions and transcription factor regulatory networks (Figure 9A). The difference in the number of cell interactions between the two groups was obtained through R‐packet CellChat. It can be seen that the number of interactions between epithelial cells and other cells in the high‐risk group is significantly higher than that in the low‐risk group. Interactions between the MIF‐associated pathways and CD44‐associated pathways in epithelial cells and immune cells were heavily activated (Figure 9B–D). Subsequently, a detailed analysis of differences in the transcription factors in the epithelia cells can be divided into four transcription modules. A large number of positive modules exist in the Module4 epithelia cells (Figure 9E). It was mainly reflected in the low‐risk group (Figure 9F). Specific analysis of each transcription factor module showed that the expression level of transcription factor CDK2 was significantly different in the high‐risk group (Figure 9G).

### 3.8 Pseudo Timing Difference Between Tumour and Normal Tissue

**FIGURE 10:**
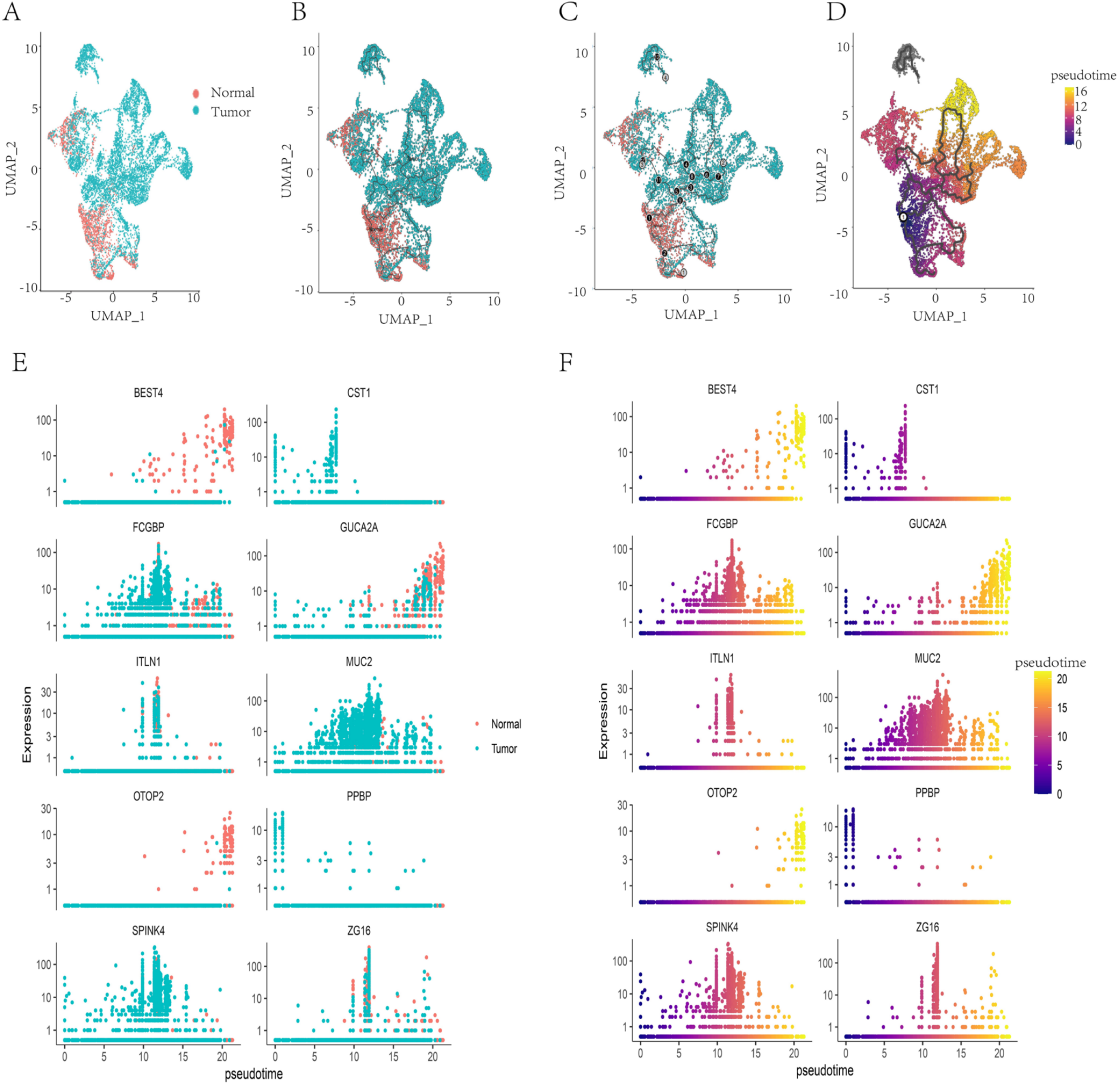
Pseudotime analysis of the epithelial cells. (A–D) Trajectory analysis shows dynamic process of the epithelial cells in normal tissue and tumour tissue. (E) Pseudotemporal cell ordering of the top 10 genes in different tissue types along differentiation trajectories. (F) Tissue types are indicated from teal to light red. Pseudotemporal cell ordering of all epidermal cell types along differentiation trajectories. Pseudotime is depicted from dark purple to light yellow.

**FIGURE 11:**
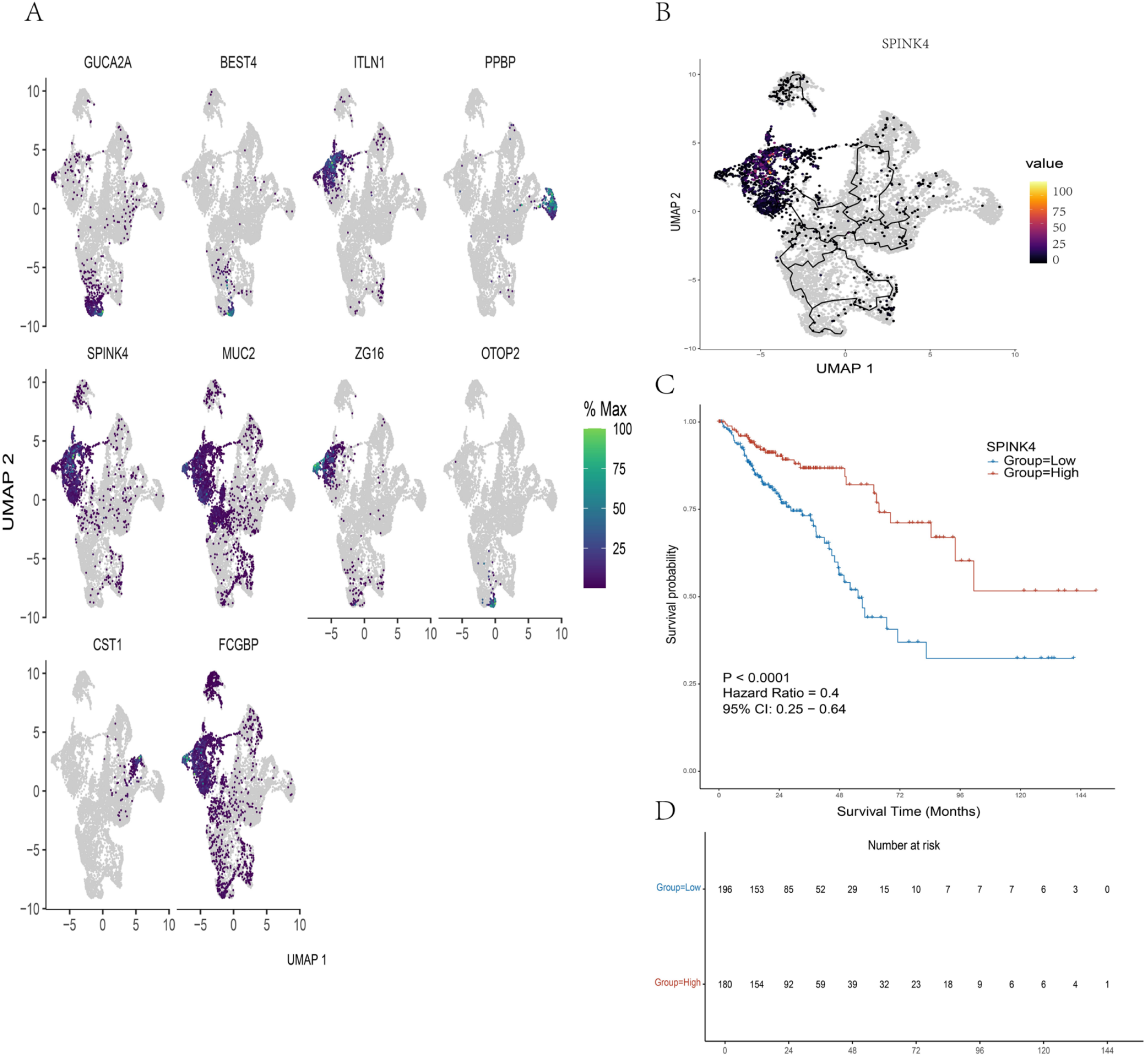
Pseudotime analysis of the epithelial cells. (A) UMAP plots showing the top 10 representative genes from pseudotemporal analysis. The colour key from dark blue to light green indicates gene expression levels from low to high. (B) UMAP plots showing the pseudotemporal trajectory of the SPINK4 gene. The colour key from dark purple to light yellow indicates gene expression levels from low to high. (C, D) The Kaplan–Meier curves showing the gene of SPINK4 in high expression group and low expression group in CRC patients.

It is still unknown what changes occur in epithelial cells from normal tissue to tumour tissue. Here, we return to the comparative analysis between the two tissues, and use R package monocle3 (version 1.3.7) to analyse the quasi‐temporal trajectory of epithelial cells from normal tissue to tumour tissue. Pseudo‐time sequence analysis confirmed that the tissue distribution of epithelial cells was consistent with the result of locus differentiation (Figure 10A–D). Then, gene module was extracted by the function find_gene_modules and the pseudo‐time sequence locus changes of the 10 genes with the greatest differences were obtained (Figure 10E,F). ‘CST1’, ‘FCGBP’, ‘GUCA2A’, ‘ITLN1’, ‘ITLN1’, ‘OTOP2’, ‘PPBP’, ‘SPINK4’, ‘SPINK4’ showed the greatest difference in the pseudosequence. Through umap visual analysis (Figure 11A), some genes were concentrated in normal tissues (e.g., SPINK4 and ITLN1), some genes were concentrated in tumour tissues (such as PPBP), and Univariate Cox regression analysis of all genes in the TCGA database revealed that SPINK4 expression was significantly associated with improved overall survival, indicating a protective prognostic role—findings detailed in Tables S5 and S6, and its expression was mainly concentrated in normal tissues according to its simulated temporal trajectory. The expression of SPINK4 gradually decreased with tumour progression (Figure 11B). The survival curve in the TCGA database proved that the survival rate of the group with high expression level of SPINK4 was significantly higher than that of the group with low expression level, indicating protective significance (Figure 11C,D).

### 3.9 PCBP3 and NGRN Increased Cell Proliferation and Invasion of HCT Cells

**FIGURE 12:**
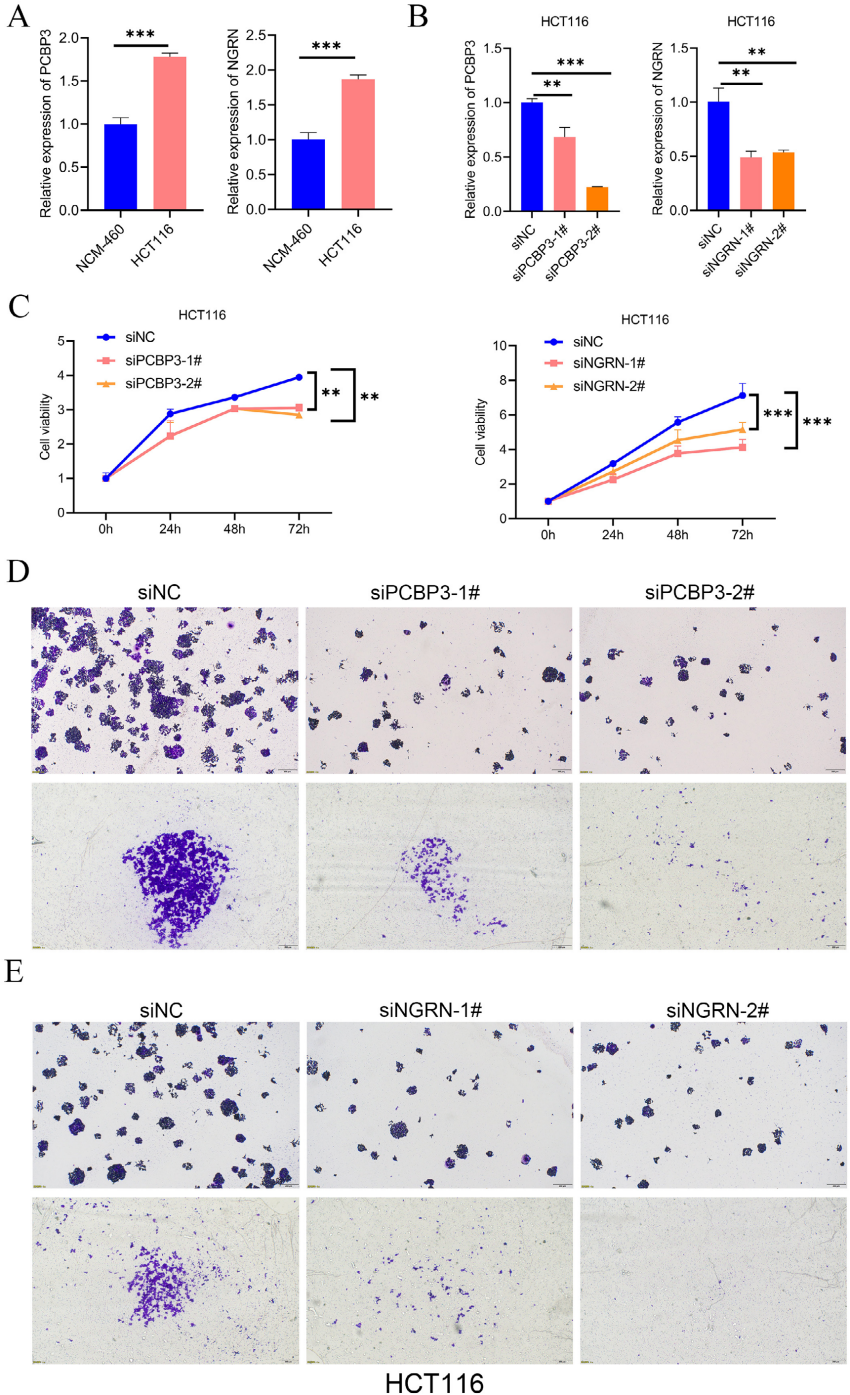
PCBP3 and NGRN promoted the proliferation and invasion of HCT116 cell. (A) qRT‐PCR to demonstrate the mRNA expression levels of PCBP3 and NGRN in NCM‐460 and HCT116 cells. (B) qRT‐PCR was conducted to detect the mRNA expression levels of PCBP3 and NGRN in HCT116 cells with PCBP3 and NGRN knockdown. The proliferation and invasion abilities of HCT116 cells are assessed through (C) CCK‐8, (D, E) colony formation and Transwell assays. NC represents the negative control group; siPCBP3‐1#, siPCBP‐2#, siNGRN‐1# and siNGRN‐2# represent the PCBP3 and NGRN knockdown groups. **p < 0.01 and ***p < 0.001 vs. NC group.

The in vitro experiments demonstrated that the mRNA expression levels of PCBP3 and NGRN were significantly higher in HCT116 cells than in NCM‐460 cells (Figure 12A). qRT‐PCR further confirmed effective knockdown of PCBP3 and NGRN in HCT116 cells after transfection with the corresponding siRNAs (Figure 12B). Consistent with these findings, the CCK‐8 assay showed that silencing PCBP3 or NGRN significantly inhibited the proliferation of HCT116 cells at 24, 48 and 72 h compared with the negative control group (Figure 12C). In addition, colony formation and Transwell assays demonstrated that knockdown of either gene markedly reduced the clonogenic and invasive capacities of HCT116 cells (Figure 12D,E). These results indicate that PCBP3 and NGRN promote malignant phenotypes in colorectal cancer cells.

To further explore the downstream mechanism, Western blot analysis was performed in HCT116 cells transfected with siNC, siPCBP3‐1#, siPCBP3‐2#, siNGRN‐1# and siNGRN‐2#. As shown in Figure S1, silencing PCBP3 or NGRN reduced the corresponding target protein levels and was accompanied by decreased expression of p‐PI3K and p‐AKT, whereas β‐actin remained stable as the loading control. These findings suggest that PCBP3 and NGRN may promote colorectal cancer progression, at least in part, through activation of the PI3K/AKT signalling pathway, thereby providing preliminary mechanistic evidence beyond the phenotypic observations.

## 4 Discussion

Colorectal Cancer (CRC) is one of the most common malignant tumours in the world, with a high incidence and mortality [39, 40, 41]. The prognosis of colorectal cancer is closely related to the stage at diagnosis. Patients diagnosed early (stage I and II) have a higher 5‐year survival rate of more than 90%. The 5‐year survival rate was significantly lower in patients with advanced stages (III and IV). Therefore, the development of new predictive biomarkers for colorectal cancer shows important clinical value in improving early diagnosis of colorectal cancer, optimizing treatment strategies, and improving patient outcomes [42]. Abnormal RNA metabolism plays an important role in the development and progression of colorectal cancer. RNA metabolism abnormalities include dysregulation of RNA synthesis, processing, stability and degradation, which can affect gene expression and function of tumour cells [43, 44, 45, 46]. Mutations or abnormal expression of splicing factors lead to mRNA splicing errors, produce abnormal proteins, and promote tumourigenesis. For example, overexpression of SRSF1 is associated with the development and progression of multiple tumours [47, 48, 49, 50]. m6A modification of RNA is often abnormal in tumours, affecting RNA stability and translation efficiency [51, 52, 53]. For example, abnormal expression of METTL3 [54, 55, 56] and METTL14 [57, 58, 59, 60] has been associated with the development and progression of multiple tumours. miRNA expression is often dysregulated in tumours, affecting the degradation or translation of target mRNA, and thus regulating the expression of tumour‐related genes. For example, overexpression of miR‐21 [61, 62] is associated with the occurrence and development of multiple tumours. In light of this, we aimed to investigate the potential significance of RMRGs in colorectal cancer and their potential as therapeutic targets through single‐cell sequencing and transcriptomics.

In the present study, we identified seven major cell clusters [63], in colorectal cancer tissues and found that epithelial cells were enriched in tumour tissues compared with normal tissues. Further CNV analysis indicated that specific epithelial subclusters exhibited markedly higher CNV scores, supporting their malignant characteristics. By integrating single‐cell upregulated genes with differentially expressed genes from the TCGA cohort, we identified PCBP3 and NGRN as the genes with the strongest prognostic relevance. The resulting two‐gene risk model showed favourable predictive performance in the TCGA, GSE17536 and GSE38832 cohorts, supporting the robustness of this signature. Moreover, the newly added HPA‐based immunohistochemical validation further demonstrated that both PCBP3 and NGRN were detectable at the protein level in colorectal cancer tissues, thereby extending the evidence beyond transcriptomic datasets and strengthening the biological plausibility of these two genes as candidate prognostic biomarkers in colorectal cancer. Our immune‐infiltration analysis further suggested that the PCBP3/NGRN‐related signature is closely associated with the tumour immune microenvironment, particularly with differences in Macrophages M0, Macrophages M2, and NK‐cell‐related features, implying a possible role in immune escape and microenvironmental remodelling in colorectal cancer [64]. Functionally, our in vitro experiments showed that knockdown of PCBP3 and NGRN significantly suppressed the proliferation and invasion of HCT116 cells. Importantly, the newly added Western blot results showed that silencing either gene was accompanied by reduced p‐PI3K and p‐AKT expression, suggesting that PCBP3 and NGRN may contribute to colorectal cancer progression, at least in part, through activation of the PI3K/AKT signalling pathway. These findings extend the study beyond transcriptomic association and phenotypic observation by providing preliminary mechanistic support. Another important finding of this study is the potential relationship between the PCBP3/NGRN‐related signature and immunotherapy‐relevant immune status. In the revised manuscript, we incorporated an IPS‐based supplementary analysis, which showed that the IPS_High group exhibited significantly lower PCBP3 and NGRN expression, lower infiltration of Macrophages M0/M2, and higher levels of activated NK cells. These findings suggest that lower PCBP3/NGRN expression may be associated not only with altered immune infiltration, but also with a more immunologically active tumour microenvironment and a potentially more favourable immunotherapy‐related immune status [65, 66, 67]. Thus, the translational significance of the PCBP3/NGRN‐related signature may extend beyond prognosis to possible stratification of immune responsiveness in colorectal cancer [68]. Nevertheless, several limitations should still be acknowledged. First, although the addition of HPA‐based immunohistochemistry improves protein‐level support for the model, validation in larger independent institutional cohorts and prospective clinical samples is still needed. Second, although the newly added Western blot results implicate the PI3K/AKT pathway, the current mechanistic evidence remains preliminary and does not yet fully explain how PCBP3 and NGRN regulate RNA metabolism, nor whether the previously identified MIF/CD44‐related intercellular communication axis is functionally downstream of these genes. Third, although the supplementary IPS‐based analysis improves the clinical relevance of the immune findings, it remains a computational and indirect evaluation. Therefore, future studies should combine post‐knockdown transcriptomic profiling, pathway and rescue experiments, and validation in independent immunotherapy‐treated colorectal cancer cohorts to further clarify the mechanistic and translational significance of PCBP3 and NGRN in colorectal cancer.

## 5 Conclusion

In conclusion, our study integrated single‐cell and bulk RNA sequencing data to identify PCBP3 and NGRN as meaningful RNA metabolism‐related genes with prognostic significance in colorectal cancer. We established a reliable two‐gene risk model that showed favourable predictive performance in the TCGA cohort and independent GEO datasets. The newly added HPA immunohistochemical analysis further supported the expression of PCBP3 and NGRN at the protein level in colorectal cancer tissues. Functional experiments demonstrated that knockdown of PCBP3 and NGRN inhibited the proliferation and invasion of HCT116 cells, while the newly added Western blot results suggested that these effects may be mediated, at least in part, through the PI3K/AKT signalling pathway. In addition, the supplementary IPS‐based analysis indicated that lower PCBP3/NGRN expression was associated with lower Macrophages M0/M2 infiltration and higher activated NK‐cell levels, suggesting that these genes may also be linked to immunotherapy‐related immune status. Collectively, these findings support PCBP3 and NGRN as potential prognostic biomarkers and candidate therapeutic targets in colorectal cancer, while also highlighting their possible relevance to tumour immune regulation and immunotherapy responsiveness. Further validation in larger clinical cohorts and mechanistic studies will be required to confirm these findings and promote their translational application.

## Author Contributions

**Fandong Kong:** conceptualization, methodology, writing – original draft, formal analysis. **Qiantao Ye:** methodology. **Shuguang Su:** methodology. **Hanpeng Du:** writing – review and editing, data curation, resources. **Feilong Chen:** methodology. **Ronghua Yang:** writing – review and editing, data curation, funding acquisition. **Xuewei Zhang:** conceptualization, methodology, writing – original draft, formal analysis.

## Funding

This study was supported by Chongqing Traditional Chinese Medicine Inheritance and Innovation Team project (2023090006KJZX2022WJW008).

## Ethics Statement

The authors have nothing to report.

## Conflicts of Interest

The authors declare no conflicts of interest.

## Supporting information


**Table S1:** Details of the dataset.
**Table S2:** A summary of the target sequences of short hairpin RNA PCBP3 and NGRN.
**Table S3:** A summary presents reagents used in reverse transcription.
**Table S4:** A summary shows the reagents used in RT‐PCR analysis.
**Table S5:** The proposed sequential partitioning approach targets a single gene.
**Table S6:** Sequential partial multigene analysis.
**Figure S1:** Immunohistochemical validation of PCBP3 and NGRN expression in colorectal cancer tissues from the Human Protein Atlas database. Representative immunohistochemical staining images of PCBP3 and NGRN in colorectal cancer tissues were obtained from the Human Protein Atlas (HPA) database. Protein expression levels were classified as not detected, low, medium and high according to staining intensity. Brown staining represents positive expression. These results provide additional protein‐level evidence supporting the potential clinical significance of PCBP3 and NGRN in colorectal cancer.
**Figure S2:** Western blot validation of PCBP3/NGRN knockdown and associated changes in PI3K/AKT signalling in HCT116 cells. Representative Western blot bands of PCBP3, NGRN, p‐PI3K, p‐AKT and β‐actin in HCT116 cells transfected with siNC, siPCBP3‐1#, siPCBP3‐2#, siNGRN‐1# and siNGRN‐2#.
**Figure S3:** IPS‐related immune characteristics associated with PCBP3/NGRN expression in colorectal cancer. Comparison of PCBP3 expression, NGRN expression, Macrophages M0, Macrophages M2, and activated NK cells between the IPS_Low and IPS_High groups in the TCGA colorectal cancer cohort. The IPS_High group exhibited significantly lower PCBP3 expression (*p* = 0.0026) and NGRN expression (*p* = 0.011), together with lower infiltration of Macrophages M0 (*p* = 1e‐05) and Macrophages M2 (*p* = 0.0067), and higher levels of activated NK cells (*p* = 0.0032). These findings suggest that lower PCBP3/NGRN expression may be associated with a more immunologically active tumour microenvironment and a potentially more favourable immunotherapy‐related immune status in colorectal cancer.

## Data Availability

The data that support the findings of this study are openly available in TCGA database and GEO database at https://www.cancer.gov/ccg/research/genome‐sequencing/tcga.
