# Supplementary material for: RNA Metabolism Genes as Prognostic Biomarkers and Therapeutic Targets in Colorectal Cancer Based on the Analysis of Single‐Cell and Bulk‐RNA Sequencing Data
Source: J Cell Mol Med. 2026 Jun 10;30(11):e71236. doi: 10.1111/jcmm.71236 (PMC13253610; doi:10.1111/jcmm.71236)
Supplement: Supplementary file 1 — Table S1: Details of the dataset. Table S2: A summary of the target sequences of short hairpin RNA PCBP3 and NGRN. Table S3: A summary presents reagents used in reverse transcription. Table S4: A summary shows the reagents used in RT‐PCR analysis. Table S5: The proposed sequential partitioning approach targets a single gene. Table S6: Sequential partial multigene analysis. Figure S1: Immunohistochemical validation of PCBP3 and NGRN expression in colorectal cancer tissues from the Human Protein Atlas database. Representative immunohistochemical staining images of PCBP3 and NGRN in colorectal cancer tissues were obtained from the Human Protein Atlas (HPA) database. Protein expression levels were classified as not detected, low, medium and high according to staining intensity. Brown staining represents positive expression. These results provide additional protein‐level evidence supporting the potential clinical significance of PCBP3 and NGRN in colorectal cancer. Figure S2: Western blot validation of PCBP3/NGRN knockdown and associated changes in PI3K/AKT signalling in HCT116 cells. Representative Western blot bands of PCBP3, NGRN, p‐PI3K, p‐AKT and β‐actin in HCT116 cells transfected with siNC, siPCBP3‐1#, siPCBP3‐2#, siNGRN‐1# and siNGRN‐2#. Figure S3: IPS‐related immune characteristics associated with PCBP3/NGRN expression in colorectal cancer. Comparison of PCBP3 expression, NGRN expression, Macrophages M0, Macrophages M2, and activated NK cells between the IPS_Low and IPS_High groups in the TCGA colorectal cancer cohort. The IPS_High group exhibited significantly lower PCBP3 expression (p = 0.0026) and NGRN expression (p = 0.011), together with lower infiltration of Macrophages M0 (p = 1e‐05) and Macrophages M2 (p = 0.0067), and higher levels of activated NK cells (p = 0.0032). These findings suggest that lower PCBP3/NGRN expression may be associated with a more immunologically active tumour microenvironment and a potentially more favourable immunotherapy‐related [file JCMM-30-e71236-s001.docx]

**Table S1.** Details of the dataset

|  | GSE17536 | GSE38832 | GSE200997 |
| --- | --- | --- | --- |
| Organism | Homo sapiens | Homo sapiens | Homo sapiens |
| Tissue | Colorectal tissue | Colorectal tissue | Colorectal tissue |
| Experiment type | Expression profiling by array | Expression profiling by array | Expression profiling by high throughput sequencing |
| Platforms | GPL570 | GPL570 | GPL21697 |
| Gene (number) | 22882 | 22882 | 30045 |
| Normal | 0 | 0 | 7 |
| Colorectal cancer | 177 | 122 | 16 |
| Total | 177 | 122 | 23 |

**Table S2.** *A summary of the target sequences of short hairpin RNA PCBP3 and NGRN.*

| **Gene name** | **Sense** | **Antisense** |
| --- | --- | --- |
| PCBP3-Homo-224 | GGAGUCCAAGGUCUCAAATT | UUCUGAGACCUUGGACUCCTT |
| PCBP3-Homo-682 | CCAUCAUCCAGUGCGUCAATT | UUGACGCACUGGAUGAUGGTT |
| NGRN-Homo-206 | CACCCUGAAACGACAGAAATT | UUUCUGUCGUUUCAGGGUGTT |
| NGRN-Homo-316 | GGUAUUUACAUGAGGAAUUTT | AAUUCCUCAUGUAAAUACCTT |

**Table S3.** *A summary presents reagents used in reverse transcription.*

| **Reagent** | **Usage** |
| --- | --- |
| RNase-free ddH_2_O | to 20 μl |
| Enzyme mix | 1 μl |
| 5 × All-in-one qRT SuperMix | 4 μl |
| RNA | Total RNA：1 pg – 1 μg |
| Program: |  |
| 50 ℃ | 15 min |
| 85 ℃ | 5 sec |

**Table S4.** *A summary shows the reagents used in RT-PCR analysis.*

| **Reagent** | **Usage - lower limit** | **Usage - upper limit** | **Final concentration** |
| --- | --- | --- | --- |
| TB Green Premix Ex Taq II (Tli RNaseH Plus) (2X) | 10 μl | 25 μl | 1× |
| PCR Forward Primer (10 μM) | 0.8 μl | 2 μl | 0.4 μM |
| PCR Reverse Primer (10 μM) | 0.8 μl | 2 μl | 0.4 μM *1 |
| ROX Reference Dye (50X) or ROX Reference Dye II (50X) | 0.4 μl | 1 μl | 1× |
| cDNA | 2 μl | 4 μl |  |
| DEPC H_2_O | 6 μl | 16 μl |  |
| Total | 20 μl | 50 μl |  |

Stage 1: Degeneration

Reps:1

95℃, 30 sec

Stage 2: PCR reaction

Reps: 40

95℃, 5 sec

60℃, 30～34 sec

Dissociation stage

**Table S5.** The proposed sequential partitioning approach targets a single gene.

| Symbol | HR | lower_95 | upper_95 | HR_95_CI | p.value | Type |
| --- | --- | --- | --- | --- | --- | --- |
| ITLN1 | 0.509 | 0.317 | 0.818 | 0.509 (0.317-0.818) | 0.005 | Protective |
| GUCA2A | 0.585 | 0.37 | 0.924 | 0.585 (0.37-0.924) | 0.021 | Protective |
| BEST4 | 1.104 | 0.718 | 1.697 | 1.104 (0.718-1.697) | 0.653 | NS |
| PPBP | 1.069 | 0.694 | 1.648 | 1.069 (0.694-1.648) | 0.762 | NS |
| SPINK4 | 0.401 | 0.253 | 0.636 | 0.401 (0.253-0.636) | 0 | Protective |
| MUC2 | 0.632 | 0.402 | 0.995 | 0.632 (0.402-0.995) | 0.047 | Protective |
| ZG16 | 0.559 | 0.35 | 0.891 | 0.559 (0.35-0.891) | 0.015 | Protective |
| OTOP2 | 0.687 | 0.436 | 1.081 | 0.687 (0.436-1.081) | 0.104 | NS |
| CST1 | 1.176 | 0.76 | 1.819 | 1.176 (0.76-1.819) | 0.466 | NS |
| FCGBP | 0.495 | 0.309 | 0.794 | 0.495 (0.309-0.794) | 0.004 | Protective |

**Table S6.** Sequential partial multigene analysis.

| Symbol | HR | lower_95 | upper_95 | HR_95_CI | p.value | Type |
| --- | --- | --- | --- | --- | --- | --- |
| ITLN1 | 0.897 | 0.434 | 1.857 | 0.897 (0.434 - 1.857) | 0.771 | NS |
| GUCA2A | 0.783 | 0.456 | 1.345 | 0.783 (0.456 - 1.345) | 0.376 | NS |
| BEST4 | 1.239 | 0.793 | 1.934 | 1.239 (0.793 - 1.934) | 0.346 | NS |
| PPBP | 1.118 | 0.712 | 1.756 | 1.118 (0.712 - 1.756) | 0.628 | NS |
| SPINK4 | 0.438 | 0.217 | 0.882 | 0.438 (0.217 - 0.882) | 0.021 | Protective |
| MUC2 | 1.525 | 0.766 | 3.034 | 1.525 (0.766 - 3.034) | 0.229 | NS |
| ZG16 | 1.04 | 0.527 | 2.05 | 1.04 (0.527 - 2.05) | 0.91 | NS |
| OTOP2 | 0.974 | 0.575 | 1.651 | 0.974 (0.575 - 1.651) | 0.922 | NS |
| CST1 | 1.146 | 0.732 | 1.795 | 1.146 (0.732 - 1.795) | 0.55 | NS |
| FCGBP | 0.689 | 0.315 | 1.506 | 0.689 (0.315 - 1.506) | 0.351 | NS |

**
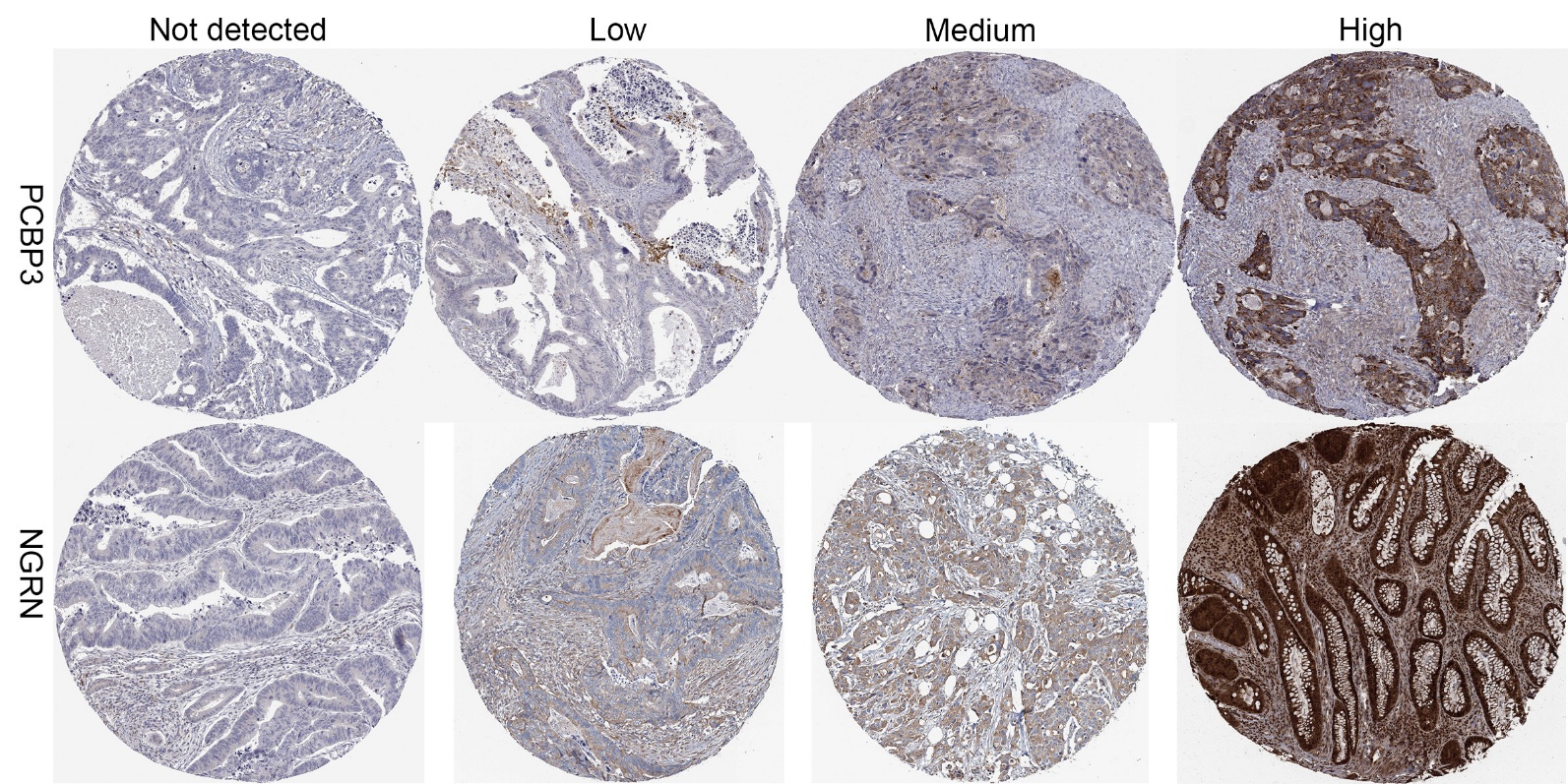
**

**Figure S1.** Immunohistochemical validation of PCBP3 and NGRN expression in colorectal cancer tissues from the Human Protein Atlas database. Representative immunohistochemical staining images of PCBP3 and NGRN in colorectal cancer tissues were obtained from the Human Protein Atlas (HPA) database. Protein expression levels were classified as not detected, low, medium, and high according to staining intensity. Brown staining represents positive expression. These results provide additional protein-level evidence supporting the potential clinical significance of PCBP3 and NGRN in colorectal cancer.


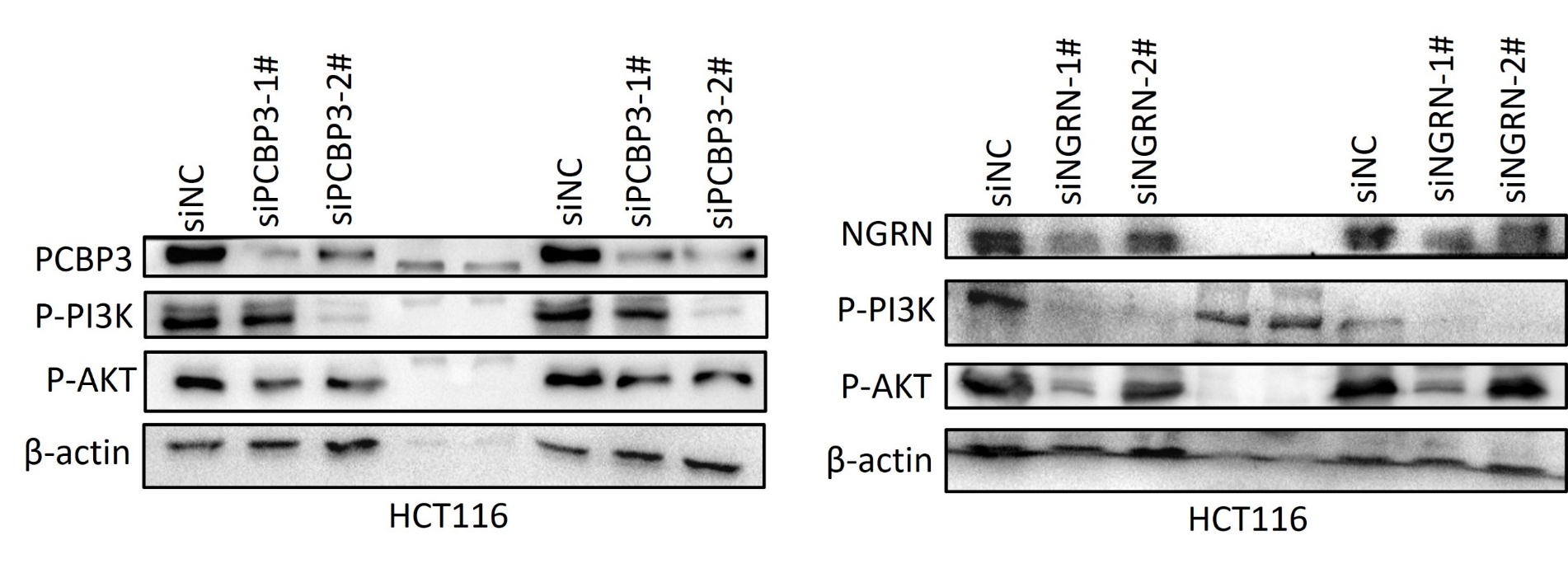


**Figure S2.** Western blot validation of PCBP3/NGRN knockdown and associated changes in PI3K/AKT signaling in HCT116 cells. Representative Western blot bands of PCBP3, NGRN, p-PI3K, p-AKT, and β-actin in HCT116 cells transfected with siNC, siPCBP3-1#, siPCBP3-2#, siNGRN-1#, and siNGRN-2#.


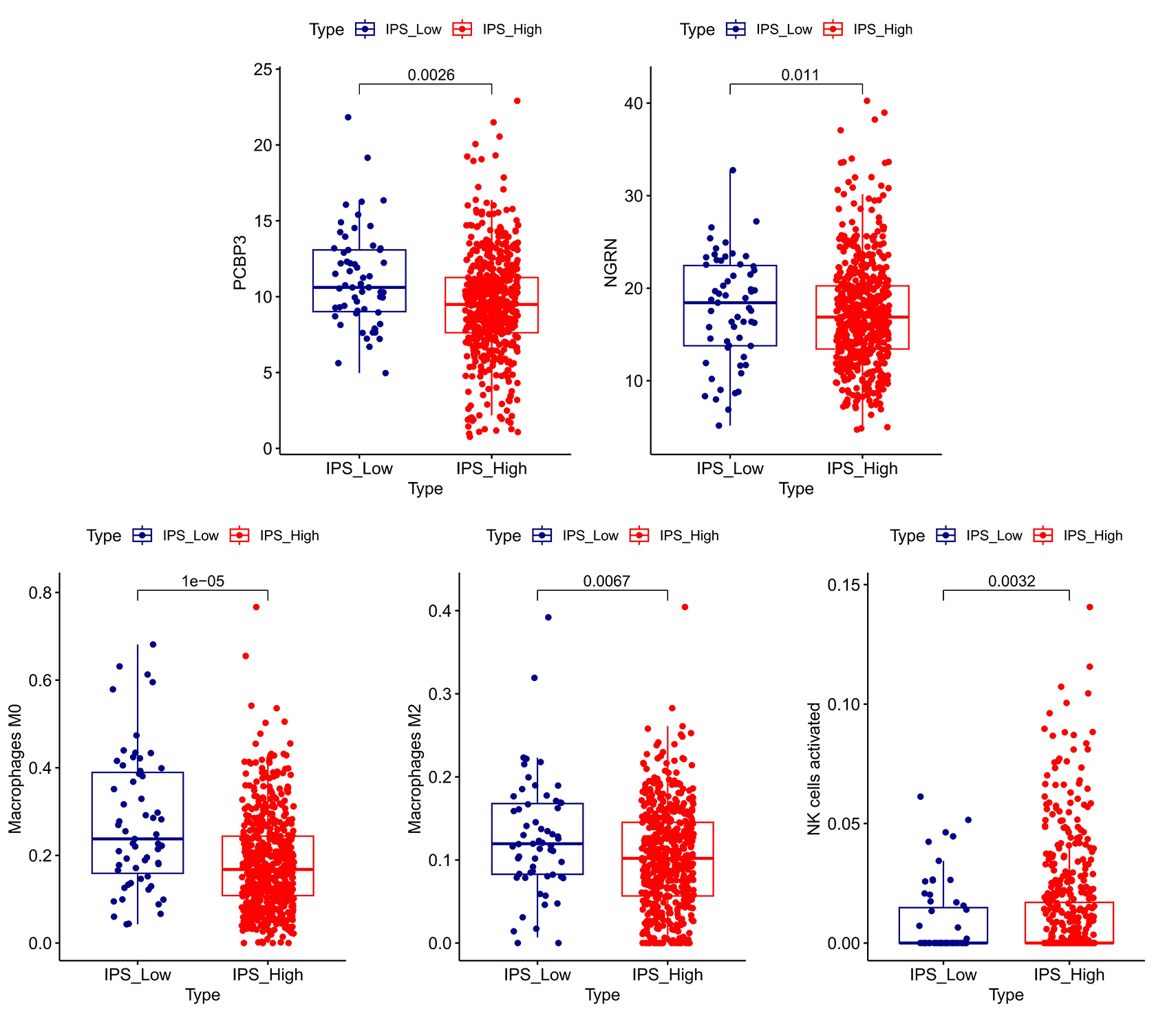


**Figure S3.** IPS-related immune characteristics associated with PCBP3/NGRN expression in colorectal cancer.

Comparison of PCBP3 expression, NGRN expression, Macrophages M0, Macrophages M2, and activated NK cells between the IPS_Low and IPS_High groups in the TCGA colorectal cancer cohort. The IPS_High group exhibited significantly lower PCBP3 expression (P = 0.0026) and NGRN expression (P = 0.011), together with lower infiltration of Macrophages M0 (P = 1e−05) and Macrophages M2 (P = 0.0067), and higher levels of activated NK cells (P = 0.0032). These findings suggest that lower PCBP3/NGRN expression may be associated with a more immunologically active tumor microenvironment and a potentially more favorable immunotherapy-related immune status in colorectal cancer.
